# Porous Single-Crystalline Monolith to Enhance Catalytic Activity and Stability

**DOI:** 10.34133/2022/9861518

**Published:** 2022-07-05

**Authors:** Xiaoyan Yu, Fangyuan Cheng, Xiuyun Duan, Kui Xie

**Affiliations:** ^1^Key Laboratory of Optoelectronic Materials Chemistry and Physics, Fujian Institute of Research on the Structure of Matter, Chinese Academy of Sciences, Fuzhou, Fujian 350002, China; ^2^University of Chinese Academy of Sciences, Beijing 100049, China; ^3^Fujian Science & Technology Innovation Laboratory for Optoelectronic Information of China, Fuzhou, 350108 Fujian, China; ^4^Key Laboratory of Design & Assembly of Functional Nanostructures, Fujian Institute of Research on the Structure of Matter, Chinese Academy of Sciences, Fuzhou, Fujian 350002, China

## Abstract

Engineering the catalytic activity and stability of materials would require the identification of the structural features that can tailor active sites at surfaces. Porous single crystals combine the ordered lattice structures and disordered interconnected pores, and they would therefore provide the advantages of precise structure features to identify and engineer the active sites at surfaces. Herein, we fabricate porous single-crystalline vanadium nitride (VN) at centimeter scale and further dope Fe (Fe_0.1_V_0.9_N) and Co (Co_0.1_V_0.9_N) in lattice to engineer the active sites at surface. We demonstrate that the active surface is composed of unsaturated coordination of V-N, Fe-N, and Co-N structures which lead to the generation of high-density active sites at the porous single-crystalline monolith surface. The interconnected pores aid the pore-enhanced fluxion to facilitate species diffusion in the porous architectures. In the nonoxidative dehydrogenation of ethane to ethylene, we demonstrate the outstanding performance with ethane conversion of 36% and ethylene selectivity of 99% at 660°C. Remarkably stability as a result of their single-crystalline structure, the monoliths achieve the outstanding performance without degradation being observed even after 200 hours of a continuous operation in a monolithic reactor. This work not only demonstrates the effective structural engineering to simultaneously enhance the stability and overall performance for practically useful catalytic materials but also provide a new route for the element doping of porous single crystals at large scale for the potential application in other fields.

## 1. Introduction

Improving catalytic materials' activity and stability would require the identification of the precise structural features that can facilitate the creation and engineering of active sites at surfaces [1–3]. The precise structure information at the depth from the top layer to sub-nanometer layer provides the fundamental basis to identify and engineer the coordination structure of the active sites at the surface of catalytic materials. Single-crystalline materials would confine the well-defined structures at the surface layer by the long-range ordering of crystal lattice. The coordination structures of active sites would therefore be created and tailored by controlling the structures of the single-crystalline materials. The preference of single-crystalline materials is mainly focused on the dimension at nanoscale, in the form of nanocrystal, to create and engineer the precise coordination structure of active surface to enhance the catalytic activity. Single-crystalline materials at nanoscale would therefore maintain the high catalytic activity, but they suffer from the thermal instability especially at high operation temperature. Bulk single-crystalline materials at macroscale with higher thermal stability could provide the advantages of long-range ordering of crystal lattice to create and engineer the coordination structure at surface layer, but their surface areas are quite limited to host the chemical reactions. They are rarely used in catalysis and normally demonstrate negligible catalytic activity even though they present high thermal stability in contrast to nanocrystals. Therefore, single-crystalline materials at macroscale with high porosity provide an alternative to synergistically engineer the catalytic activity and thermal stability.

Porous single-crystalline materials synergistically incorporate ordered crystal lattices and disordered interconnected pores in bulk-porous architecture at macroscale, which therefore demonstrates the combined advantages of porous materials and single-crystalline materials. The long-range ordering of crystal lattice provides the huge potential to identify and engineer the well-defined structure features to create active surface regions [4, 5]. Porous single-crystalline materials at macroscale are therefore more thermally stable than single crystal at nanoscale, but the single-crystalline skeleton at nanoscale would demonstrate the comparable catalytic activity in contrast to nanocrystals. Porous single-crystalline materials have the interconnected pores in the porous architectures, which leads to the comparable advantage of porosity in contrast to porous materials. The porous architectures not only provide high surface areas to accommodate the chemical reactions but also facilitate species diffusion through three-dimensional percolation. The interaction of adsorbents with active surface in catalysis could be therefore tailored by the well-defined coordination structures at surface and the enhanced species diffusion in the porous single-crystalline architecture. Porous single-crystalline materials at macroscale are readily machinable into complex monolithic architectures in the form of catalytic unit and reactor to well suit the current infrastructures in chemical industry.

Transition metal nitride like vanadium nitride (VN) is widely used for catalytic dehydrogenation of alkane to produce olefin. They possess unique physicochemical properties that are due to the presence of nitrogen atoms at the interstitial sites [6–8]. The unsaturated V-N coordination structure would produce active sites at the well-defined surfaces to tailor the activity and stability of catalytic materials. Porous single-crystalline vanadium nitride monoliths would therefore demonstrate the advantages to create and engineer the coordination structures of active sites at the surface layers, and the porous architectures would also function in the form of monolithic reactors. In chemical industry, ethylene as an important building block can be produced from the steam cracking of ethane. In shell gas, ethane contributes to ~20% in volume, which provides the huge potential to convert ethane into high-value ethylene. The steam cracking process is energy-intensive with an operation temperature higher than 800°C, which inevitably encounters the adverse challenges including side reactions, severe coking, and CO_2_ emissions in the process [9–12]. Nonoxidative dehydrogenation of ethane to ethylene is an effective pathway to maintain high ethylene selectivity, but the ethane conversion is limited and accompanied by undesirable coke formation [13–16].

Herein, we fabricate the porous single-crystalline vanadium nitride (VN) monolith at 1 cm scale to create the well-defined active surface and further dope Co and Fe in lattice to engineer the coordination structures of the surfaces to enhance the catalytic activity and durability of nonoxidative dehydrogenation of ethane to ethylene. In a monolithic reactor, we demonstrate the exceptionally high ethane conversion and ethylene selectivity as well as the outstanding durability even in a continuous operation of 200 hours in nonoxidative dehydrogenation process at reduced temperatures.

## 2. Results and Discussion

We fabricate porous single-crystalline (PSC) VN, Fe_0.1_V_0.9_N (FVN) and Co_0.1_V_0.9_N (CVN) monoliths at 1 cm scale by using a lattice reconstruction strategy. Parent single crystals of BiVO_4_, FeVO_4_, and CoV_2_O_6_ are directly treated in ammonia atmosphere and converted into PSC VN, PSC FVN, and PSC CVN monoliths, respectively. The average roughness of the surface of parent single crystals is polished to be below 0.3 nm to sufficiently eliminate the influence of surface instability as shown in Figure S1. Figure S2-S4 present the growth mechanism of PSC monoliths, which indicates the lattice shrinkage from parent single crystals to PSC nitride monoliths leading to the creation of porosity in single crystals. In nitriding process, the removal of Bi/O in conjunction with the formation of V-N bond in the recrystallization process leads to the growth of PSC monoliths. As shown in Figure S5-S7, the energies are less than ~6.4 eV for removing Bi, Co, and Fe in (001) BiVO_4_, (0-11) CoV_2_O_6_, and (100) FeVO_4_, while the energies are below ~5.0 eV for the replacement of O with N in (001) BiVO_4_, (0-11) CoV_2_O_6_, and (100) FeVO_4_. The reasonable energies would facilitate the growth of PSC monoliths in the nitriding process at high temperatures. In addition, the reasonable lattice mismatch below 25% finally leads to the preferential growth of PSC (220) VN, PSC (200) FVN, and PSC (200) CVN monoliths from the (001) BiVO_4_, (0-11) CoV_2_O_6_, and (100) FeVO_4_, respectively.

**Figure 1:**
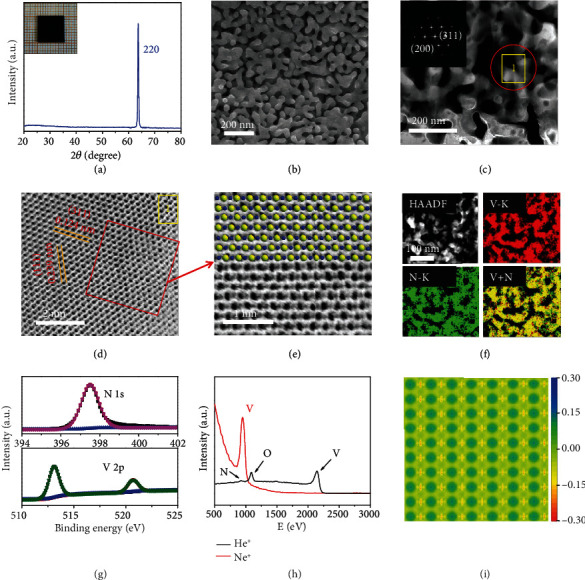
Microstructure of PSC VN monolith. (a) X-ray diffraction (XRD) and the digital image (10 × 10 × 0.5 mm). (b) SEM of porous VN single crystal with (220) facet. (c) Scanning transmission electron microscope (STEM) image of the pore framework. (d and e) High-resolution transmission electron microscope (HRTEM) and atom stacking images of VN crystal. N in blue and V in yellow. (f) HAADF image and element mapping of porous (220) VN single crystal. (g) XPS spectroscopy of porous (220) VN single crystal. (h) HS-LEIS spectra of porous VN single crystal with (220) facet. (i) Differential charge density graph of the outmost surface.

Figure 1(a) shows the X-ray diffraction (XRD) of PSC (220) VN monolith which confirms that the porous architecture is in a single-crystalline state. Figure 1(b) shows the scanning electron microscopy (SEM) picture of the PSC (220) VN monolith, which indicates porous microstructure with the pore size of about 37 nm well fit the result from Brunauer-Emmett-Teller (BET) test in Figure S8. The porosity of the PSC VN monoliths grown from BiVO_4_ is about 78%, which is mainly dominated by the structure and composition of the parent single crystals. Figure 1(c) shows the scanning transmission electron microscope (STEM) image of PSC (220) VN monolith, which again confirms the interconnected pores (30-40 nm) in the architecture. The inset shows the selected area electron diffraction (SAED) pattern of the porous architectures, which indicates the single-crystalline nature of PSC (220) VN monolith. Figure 1(d) exhibits the high-resolution Cs-corrected STEM image of PSC VN monolith. The lattice spacing of 0.239 and 0.124 nm well corresponds to the (111) and (311) lattice fringes, respectively. Figure 1(e) presents ordered atom stacking indicating the single-crystalline features in the partial enlargement of Cs-corrected STEM image. Figure 1(f) shows that the V and N are evenly distributed in the skeleton as validated by the high-angle annular dark-field (HAADF) image and elemental mapping. Figure 1(g) presents the X-ray photoelectron spectroscopy (XPS) of the PSC VN monoliths which indicates that the elements V and N exist stably in the +3 and -3 of chemical states, respectively. Figure 1(h) shows high-sensitivity low-energy ion scattering spectroscopy (HS-LEISS) of the PSC VN monoliths. Among them, He^+^ and Ne^+^ spectra are more sensitive to light and heavy elements, respectively. It implies that the top layer is composed of V even with negligible amount of adsorbed oxygen species. Figure 1(i) shows the simulated electronic structures at surfaces, which indicates the high-density active sites composed of unsaturated V-N coordination structures at the surface.

**Figure 2:**
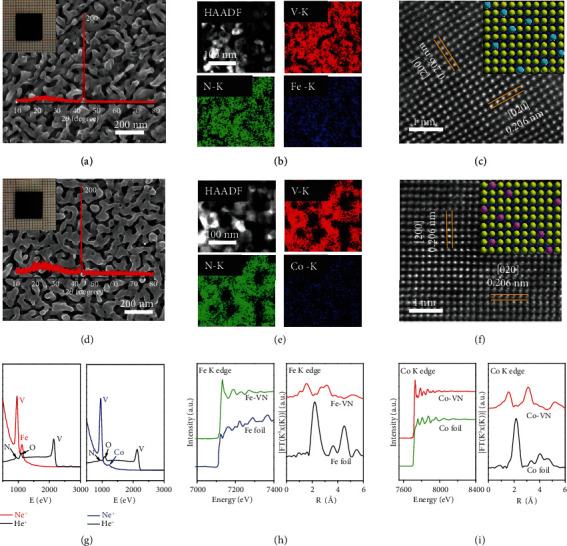
Microstructure of porous Fe_0.1_V_0.9_N and Co_0.1_V_0.9_N single crystals. (a and d) XRD pattern and SEM of porous Fe_0.1_V_0.9_N and Co_0.1_V_0.9_N single crystal with (200) facet, respectively. The digital images of a crystal (10 × 10 × 0.5 mm) in the inset. (b and e) HAADF and elemental mapping image of FVN and CVN, respectively. (c and f) Cs-HRTEM images and atomic structures of the FVN and CVN crystals. N in blue, V in yellow, Fe in blue-green, and Co in pink. (g) HS-LEIS spectra of the outermost surface of FVN and CVN single crystals. (h) XANES spectra and *k* space for porous FVN single crystal and Fe foil. (i) XANES spectra and *k* space for porous CVN single crystal and Co foil.

We further dope Fe and Co in the lattice of PSC VN monoliths to engineer the active sites at surface by directly growing PSC FVN and CVN monoliths. The chemical composition of doped PSC FVN and CVN monoliths is tailored by controlling the pressure and duration time of growing conditions. Figures 2(a)–2(c) and 2(d)–2(f) show the XRD, SEM, EDX, and STEM of the PSC FVN and CVN monoliths. We observe the preferential growth of [200] orientation of the PSC FVN and CVN monoliths even from the two different parent single crystals. The Fe and Co are successfully doped at the V sites in lattice. The uniform microstructure of PSC (200) FVN monolith indicates the pore size of ~30 nm in the porous architecture. The elemental mapping image of the porous framework shows the homogeneous distribution of Fe, V, and N elements in the single-crystalline skeleton. The lattice spacing of 0.206 nm corresponds to (200) and (020) planes. Similarly, the uniform microstructure of PSC (200) CVN monolith gives the pore size of ~58 nm and the homogeneous element distribution in the skeleton the porous architecture. The *d*-spacing of 0.206 nm also well matches the (020) and (200) facets. The chemical formula of PSC FVN and CVN monoliths are confirmed to be Fe_0.1_V_0.9_N and Co_0.1_V_0.9_N in sequence as validated by the inductively coupled plasma mass spectrometry (ICP-MS) in Figure S9.

Figure 2(g) shows the HS-LEIS spectra at the surface of PSC FVN and CVN monoliths. The V and Fe are at the top layer and bonded with the beneath-layer N to form unsaturated metal-nitrogen coordination structures. Similarly, the Co and V at the top layer bond with the beneath-layer N at the surface of PSC CVN monolith. A negligible amount of adsorbed oxygen species is also observed at the surface for the PSC FVN and CVN monoliths. Figure S10 and S11 show the visualized structures of the surface of PSC FVN and CVN monoliths, which exhibits the charge density difference diagrams at the unsaturated metal-nitrogen coordination structures. Compared with V, the charge density difference around Fe and Co changes into lower state, indicating the smaller electronegativity of Fe and Co in contrast to V in lattice. Figure S12 shows the density of states for VN, FVN, and CVN, which indicates the similar electronic structure even though the Fe and Co are doped into lattice. Figure S13 shows the XPS of PSC FVN and CVN monoliths which indicates the formation of Fe-N and Co-N bonds [17, 18]. We further acquire the extended X-ray absorption fine structure spectroscopy (EXAFS) of PSC FVN and CVN monoliths to obtain the X-ray absorption near-edge structure spectroscopy (XANES) and Fourier transforms (FTs) of K edges (Fe and Co). We infer the Fe-N and Fe-V coordination numbers in Table S1 by fitting the EXAFS data in Figures 2(h) and 2(i). Localized metallic Fe-N and Co-N bonds can be observed in PSC FVN and CVN samples at around 3.6 and 4.1 Å, respectively. The coordination properties are examined using wavelet transform of the Fe and Co K edge-extended X-ray absorption fine structure (EXAFS), as shown in Figure S14 and S15. The coordination properties of Fe and Co in FVN and CVN are quite different from those in foil Fe and Co, with the strength maxima of Fe-N and Co-N coordination being 3.6 and 4.1 Å, respectively. This result indicates the existence of Fe-N and Co-N coordination values in the confined system in good agreement. Therefore, the quantitative doping of Fe and Co elements can form an electronic structure in VN with Fe-N and Co-N bonds, thereby promoting the activation of ethane and enhancing the catalytic activity.

**Figure 3:**
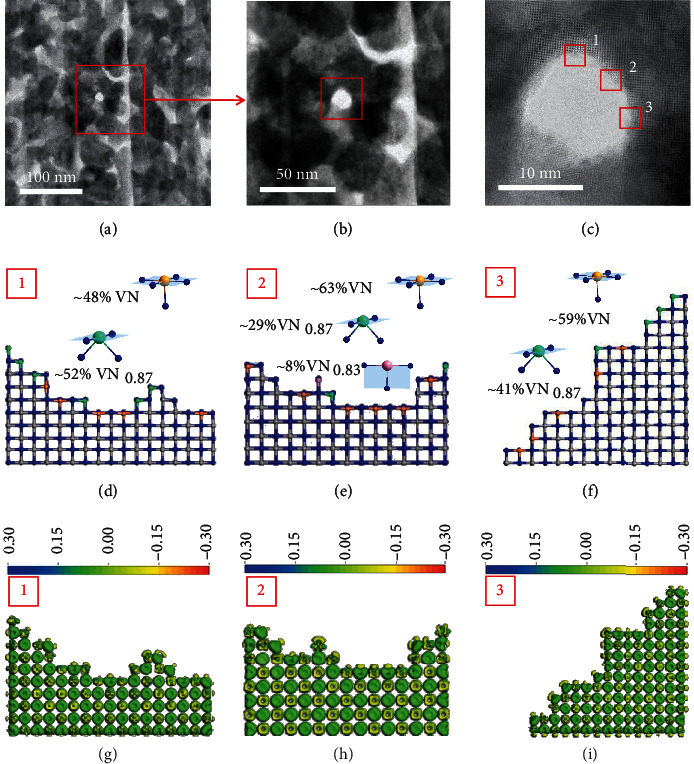
Identification of pore structure of porous VN single crystal. (a–c) STEM images with different scale bars (scale bar at 100 nm for (a), 50 nm for (b), and 10 nm for (c)). (d–f) Unsaturated V-N bonds at pore surfaces. Gray balls are V atoms, and blue balls are N atoms. (g–i) Differential charge density at the twisted surface of porous VN single crystal in (d), (e), and (f), respectively.

Figures 3(a)–3(c) demonstrate the morphology of the PSC VN monolith with a pore size of approximately 10 nm. The top layer of the PSC VN monoliths is mainly V atoms, leading to the unsaturated coordination of V-N structures at surfaces around the pores. Based on these results, we visualize the surface structures around the pores where the regions 1, 2, and 3 are denoted in Figure 3(c). Figures 3(d)–3(f) and Figure S16 present the coordination structures at the denoted areas. The coordination structures at regions 1, 2 and 3 are calculated to be 48% VN+52% VN_0.87_, 63% VN+29% VN_0.87_+8% VN_0.83_, and 59% VN+41% VN_0.87_, respectively. The surface around the pores is composed of unsaturated V-N coordination structures. Although the V-N coordination structure dominates the well-defined surfaces, we still observe ~30-50% of the highly unsaturated coordination structure including VN_0.87_ and VN_0.83_ at surfaces. We further visualize the electronic states of regions 1, 2, and 3 at the surfaces in Figures 3(g)–3(i) and show that the well-defined surface is electron-deficient with obvious charge transfer from V atoms to N atoms leading to the high density of active sites. The unsaturated V-N structures could be high-density active sites responsible for the catalytic activity for the activation of C-H bond in ethane dehydrogenation.

**Figure 4:**
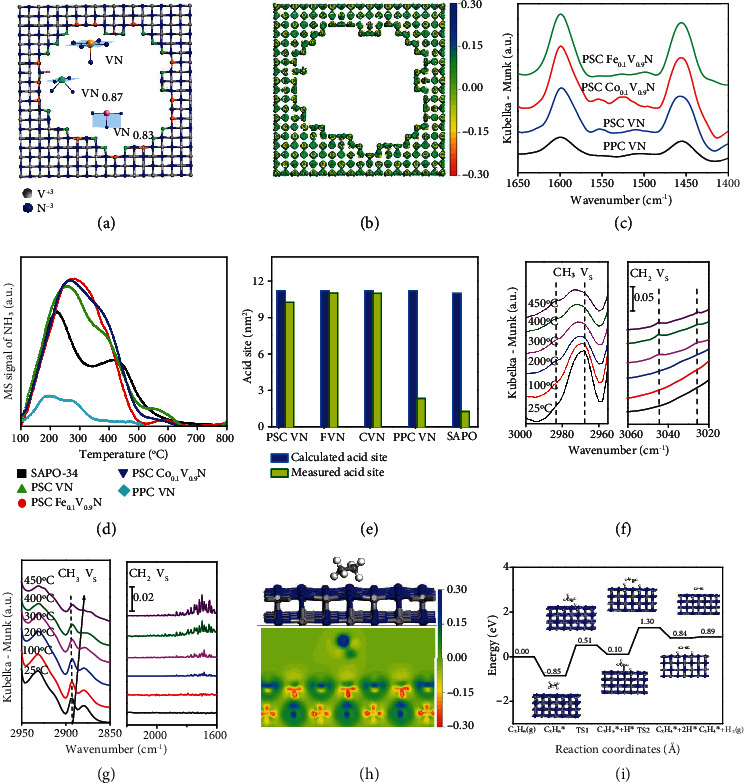
Mechanism of nonoxidative ethane dehydrogenation. (a) Unsaturated VN, VN_0.87_, and VN_0.83_ sites at the twisted surface of porous VN single crystal and (b) the charge density difference. (c) FTIR spectra of adsorbed pyridine. (d) NH_3_-TPD profiles of PSC, porous polycrystalline (PPC) catalysts, and molecular sieves sample (SAPO-34). (e) The density of Lewis acid site of different catalysts. (f and g) *In situ* FTIR of C-H bond stretching vibration and C=C bond stretching vibration on porous VN single crystals. (h) The adsorption and activation process of ethane molecules on (200) VN. (i) Potential energy profile for the ethane dehydrogenation on VN (200) surface (V in silver, N in blue, C in gray, and H in white).

We further simulate the surface structures around the pores and present the high-density active sites composed of unsaturated V-N coordination structures in Figures 4(a) and 4(b). The charge density difference diagram indicates the electron-deficient surface around the pores in the PSC VN monoliths. In the pores, the coordination structures are composed of 50%-70% VN and 30%-50% VN_0.87_/VN_0.83_ at surface. We conduct the test of Fourier transform infrared spectroscopy (FTIR) of pyridine adsorption to distinguish the acid nature of the active sites at 150°C as shown in Figure 4(c). The surface is free of Brønsted acid sites because of the absence of the characteristic absorption band near 1540 cm^−1^ for PSC monoliths. The presence of Lewis acid sites at surface is confirmed by the well-resolved characteristic absorption bands in the range of 1440-1460 cm^−1^ and 1590-1610 cm^−1^ [19]. We conduct the test of temperature-programmed desorption of ammonia (NH_3_-TPD) experiments in Figure 4(d) to validate the acidity of the active sites and show that the desorption peak of NH_3_ at 150-350°C and 350-500°C can be attributed to the weak and moderate acid sites, respectively. We further show that the doping of Fe and Co at the site of V in lattice increases the acidity of the active sites as validated by the characteristic peak at higher temperatures. We calculate the total density of acid sites when we assume that one NH_3_ molecule adsorbs on one acid site. PSC VN, FVN, and CVN monoliths therefore demonstrate the density of acidic sites of 396, 410, and 426 *μ*mol g^−1^, respectively. In Figure 4(e) and Figure S17, it is calculated that the theoretical value of the acid site is 11.21 nm^−2^ for all the PSC nitride samples, which is well consistent with the density of metal atoms at the top layer of the porous single crystal and is ~5.5 times higher than that of the polycrystalline nitride samples. We conduct the test of *in situ* FTIR to clarify the activation of ethane as shown in Figure 4(f). We see the red shift of the peak indicating the high energy state of ethane at higher operation temperatures. Strong adsorption peaks can be observed in the infrared spectrum of 3000-2600 cm^−1^ that is associated with the C-H bond of the CH_3_ group in ethane molecules [20]. The bands at 2968 and 2983 cm^−1^ designated as C-H bond asymmetric stretching vibration of the methyl group become visible and clear even at 300°C which again validates the effective activation of ethane to ethylene at reduced temperatures. The C-H bond of CH_2_ group symmetric stretching vibration is observed at 3024 and 3045 cm^−1^ on PSC VN monoliths. In Figure 4(g), the C-H bond (CH_3_) symmetric stretching vibration and the C=C bond (CH_2_) symmetric stretching vibration again confirm the generation of ethylene at reduced temperatures. We believe that the high-density active sites would capture and activate ethane at the surface and finally dehydrogenate ethane into ethylene even only at reduced temperatures.

We perform the theoretical calculation to understand the mechanism of the activation of ethane to ethylene in Figures 4(h) and 4(i). The bond lengths of C-C and C-H in C_2_H_6_ are 1.528 and 1.099 Å for the gaseous molecule, respectively. The bond angle between C-C and C-H is 111.45-111.50°. After chemisorption on (200) VN surface, a new bond of V-H bond is formed with the length of 2.619 Å as shown in Figure 4(h), which indicates the effective chemical interaction between ethane molecules and active sites. The bond lengths of C-C and C-H change by +0.003 and+0.004 Å, respectively. The bond angle between C-C and C-H is also changing to the range of 110.95-111.87°. In the adsorption process, C and H atoms accept the electrons from the V atoms with the adsorption energy of ethane at -0.85 eV, which is favorable to the process of ethane chemisorption at the surface. These results indicate the effective activation of ethane molecules at the high-density active sites at the electron-deficient surface in the PSC VN monolith. We further calculate the transition states of ethane dehydrogenation as shown in Figure 4(i) and present that the pathway of ethane dehydrogenation: C_2_H_6_∗ → TS1 → C_2_H_5_∗ → TS2 → C_2_H_4_∗. The energy barrier of C_2_H_5_ formation is 1.36 eV with a reaction energy of 0.95 eV, and the barrier of C_2_H_4_ formation is 1.20 eV with a reaction energy of 0.74 eV. The potential barriers are less than 1.40 eV, indicating that the energy required for reactions is reasonable and the reactions are easy to proceed at the active surfaces.

**Figure 5:**
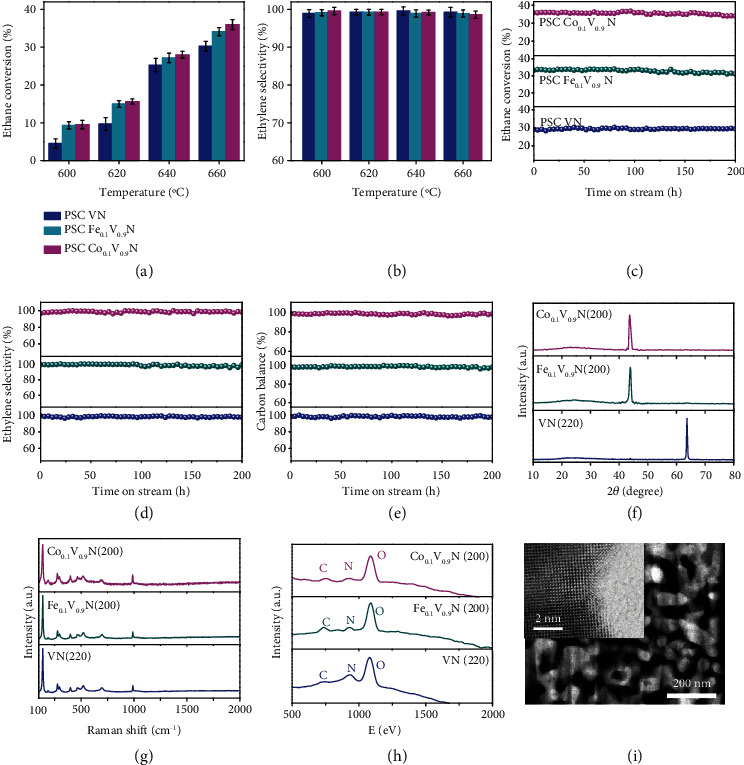
Performance of nonoxidative ethane dehydrogenation and durability. (a and b) The ethane conversion rate and ethylene selectivity using different PSC monoliths. (c–e) The durability test and carbon balance for nonoxidative ethane dehydrogenation at 660°C. (f–h) XRD, Raman spectroscopy, and HS-LEISS of PSC VN, Fe_0.1_V_0.9_N, and Co_0.1_V_0.9_N monoliths after the continuous operation of 200 hours using 10% C_2_H_6_/90% Ar at 660°C. (i) STEM and Cs-HRTEM images of PSC VN after stability test for 200 hours.

Figures 5(a) and 5(b) show the ethane conversion and ethylene selectivity for the nonoxidative dehydrogenation of ethane with PSC VN, FVN, and CVN monoliths at 600-660°C. The ethane conversion generally increases *versus* operation temperature and finally reaches about 29%, 34%, and 36% for the PSC VN, FVN, and CVN monoliths at 660°C, respectively. The ethylene selectivity still maintains at as high as 97%-99% even at the high ethane conversion for all the PSC monoliths at 600-660°C. It should be noted that the ethane conversion generally approaches the thermodynamic equilibrium which indicates the effective activation of ethane in the PSC nitride monoliths. For comparison, we prepare porous polycrystalline (PPC) VN monoliths with a pore size of ~40 nm and show that the polycrystalline framework has amorphous layer around the pores in Figure S8 and S18. Figure S19 shows the nonoxidative ethane dehydrogenation using PPC monoliths, which indicates that the catalytic performance is about 5 times lower than that of the PSC monoliths at 660°C. The well-defined surfaces composed of the unsaturated metal-nitrogen coordination structures create the high-density active sites at the clean surfaces and activate the dehydrogenation of ethane to avoid the side reaction. The moderate doping of nonprecious metal Fe and Co elements also effectively engineer the acidity of active sites and further enhance the ethane conversion and ethylene selectivity.

In Figures 5(c)–5(e), the durability test is carried out with PSC nitride monoliths at 660°C for a continuous operation of 200 hours, which shows that the ethane conversion and ethylene selectivity are generally not attenuated. The carbon balance is maintained at 95%-99% during the long-term operation.  Figure 5(f) and Figure S20 show the XRD of the PSC nitride monoliths after the long-term stability test, which confirms the absence of impurity phases and carbon species. In Figure 5(g) and Figure S21, the test of Raman spectroscopy reveals the absence of carbon deposition within the detection limit. The HS-LEISS in Figure 5(h) and Figure S22 shows a weak carbon signal by 3 KeV He^+^ ion scattering, which indicates that there are trace amounts of carbon at the top layer of the PSC monolith after the stability test. Although the performance degradation is not observed, the formation of carbon from over-cracking of ethane is still present at a trace amount. In Figure 5(i) and Figure S23, the STEM and SEM present the uniform microstructures with a pore size of ~30 nm and well-defined surface structures around the pores even after stability test. The effective structural engineering to tailor the coordination structures at the active surface simultaneously enhances the stability and activity of the PSC monoliths.

## 3. Conclusions

In conclusion, we have fabricated porous single-crystalline VN and Co-/Fe-doped VN monoliths at 1 cm scale and then identified and engineered the structural features to create well-defined active sites at surfaces to simultaneously enhance the catalytic activity and stability. The monoliths demonstrate outstanding catalytic performance with ethane conversion of 36% and ethylene selectivity of 99% at 660°C in the nonoxidative dehydrogenation of ethane. The well-defined surfaces composed of unsaturated metal-nitrogen coordination structures contribute to the effective activation of ethane, while the long-range ordering of lattice confines the active surface to deliver enhanced durability. We therefore show the outstanding performance of nonoxidative dehydrogenation of ethane even after a continuous operation for 200 hours. The current work will open new pathway not only to realize the enormous potential of catalytic materials but also to enable dramatic catalytic reaction kinetics improvements across a range of research fields.

## 4. Materials and Methods

### 4.1. Growth of Porous Single-Crystalline Monoliths

The parent crystals BiVO_4_, FeVO_4_, and CoV_2_O_6_ are grown by the flux method and the Bridgman-Stockbarger method. The crystals are oriented and cut into chips with 1 × 1 cm and a thickness of 0.5 mm. The parent crystal is placed horizontally in a vacuum alumina chamber equipped with a high-precision pressure controller for the growth of porous single crystals. In order to grow PSC (220) VN monoliths from BiVO_4_ single crystal, the NH_3_ is controlled at the flow rate of 100-300 sccm at 900°C and 400-600 Torr for 50 hours. Similarly, the growth of PSC (200) VN monoliths with Fe and Co dopant from FeVO_4_ and CoV_2_O_6_ single crystals is controlled with the flow rate of NH_3_ at 200-500 sccm with 300-600 Torr at 700°C for 50 hours. The porous polycrystalline samples are also grown at atmospheric pressures with NH_3_ flow rate of 50-1000 sccm. All samples are collected at room temperature and protected in a vacuum.

### 4.2. Characterization of Porous Single-Crystalline Monoliths

Atomic force microscopy (AFM, size Icon, Brook) is used to evaluate the topographic information at the surface of parent crystals. X-ray diffraction (XRD, miniflex 600, Rigaku) is used to identify the formation and orientation of the crystal in the range of 10-80° at a scan rate of 15° min^−1^ (*λ* =1.5418 Å). Field emission scanning electron microscope (FE-SEM, SU-8010) with the acceleration voltage of 10 kV is used to observe the microscopic morphology of the PSC monoliths. Surface area and pores are analyzed by N_2_ adsorption and desorption on a porosity analyzer (BET, Tristar II 3020). The focused-ion-beam (FIB) nano-tomography (Helios 650, Zeiss Auriga) is used to prepare samples with the thickness lower than 100 nm. FIB nanoscale tomography is performed to sample slice for transmission electron microscope (TEM) characterization. Cs-TEM (FEI Titan3 G2 60-300) is used to analyze the crystal lattice structure and elemental mapping. Selected area electron diffraction (SAED) pattern is executed to clarify the crystal orientation. Raman spectrum is obtained by characterizing the samples with the 532 nm wavelength excitation (LabRAM HR Evolution, Horiba Jobin Yvon) at room temperature.

The characterization of the top layer of the PSC monoliths is performed on a high-sensitivity low-energy ion scattering spectrometer (Qtac100, ION-TOF) equipped with a dual-ring analyzer using 3000 eV He^+^ with 6000 pA current and 5000 eV Ne^+^ with a 3000 pA current as ion sources. X-ray photoelectron spectroscopy (XPS) is performed with a ESCALAB 250Xi photoelectron spectrometer equipped with an Al K*α* source. The elemental content data for the samples are collected using ICP-OES by UTLIMA-2 (JY, France). The *in situ* FTIR spectrum is obtained by characterizing the PSC monoliths in the diffuse reflectance mode of the Bruker Optic TENSOR infrared spectrometer equipped with a mercury cadmium telluride (MCT) detector at a resolution of 4 cm^−1^ over 32 scans. The PSC monoliths are firstly heated to 350°C in He gas flow for pretreatment. The test is performed after adsorbing pyridine at room temperature and then removing the physically adsorbed pyridine in a vacuum. NH_3_-TPD is measured on AutoChem II 2920 (Automatic Catalyst Characterization System). The PSC monoliths are pretreated in He gas flow at 150°C for 1 hour and then reduced to 50°C in a 10% NH_3_/He (V/V) gas flow for 1 hour. The processed PSC monoliths are purged at a temperature of 100°C and kept at the same flow rate for another 1.5 hours. The temperature is increased from 100 to 800°C at 10°C min^−1^ and kept at 800°C for 10 min. Tracking M/z = 16 segments peak signal.

### 4.3. XAFS Measurements and Analysis

The X-ray absorption find structure spectra (Fe and Co K edge) are executed by the 1W1B of Beijing Synchrotron Radiation Facility (BSRF). The operating voltage of the BSRF storage ring is 2.5 GeV, and the average current is 250 mA. The EXAFS data are obtained through Si (111) double-crystal monochromator in transmission/fluorescence mode in ionization chamber at the condition of room temperature and atmospheric pressure. The acquired EXAFS data are analyzed by the standard procedures with the ATHENA module in the IFEFFIT software packages. The *k*^3^-weighted EXAFS spectra are obtained by subtracting the post-edge background from the overall absorption and then normalizing with respect to the edge-jump step. Subsequently, *k*^3^-weighted *χ*(k) data of K-edge (Fe and Co) are Fourier transformed to real (R) space using a Hanning windows (dk = 1.0 Å^−1^) to separate the EXAFS contributions from different coordination shells. The least-square curve parameter fitting is executed by the ARTEMIS module of IFEFFIT software packages to obtain the quantitative structural parameters around central atoms [21, 22].

The density of Lewis acid sites at surface is calculated according to

(1) $$\begin{array}{c} d=\frac{D_{\text{SAPO}-34}\times A\times N_{A}}{A_{\text{SAPO}-34}\times S_{\text{BET}}}, \end{array}$$

where *d* is the density of Lewis acid site of PSC VN monoliths, *D*_SAPO−34_ is the density of acid site of standard sample SAPO-34 which is 2.55 × 10^−4^ mol/g, *A* is the integral area of the mass spectrometry signal of -NH_2_ of PSC VN monoliths harvested by NH_3_-TPD, *A*_SAPO−34_ is the integral area of mass spectrometry signal of NH_3_ of SAPO-34, *N*_*A*_ is the Avogadro's number, and *S*_BET_ is the specific surface area of VN. We calculate the density of Lewis acid sites of PSC VN monoliths: *d* = 10.25 nm^−2^. The density of V atoms at top layer of unit cell of VN with different facets is calculated according to

(2) $$\begin{array}{c} n=\frac{N}{a\times b\times \sin\left(\alpha \right)}, \end{array}$$

where *n* is the density of top layer V atoms of VN at different facet, *N* is the number of V atom at the top layer of unit cell, *a* and *b* are the cell parameters of unit cell, and *α* is the angle between *a* and *b*. We adopt the three strongest X-ray-diffraction facets of VN to calculate the average density of V atom at top layer of (200), (220), and (222) facets of VN to represent the density of top layer V atoms at surfaces. The calculation is *n*_[200]_ = 2/(0.412 nm × 0.412 nm × sin (90°)) = 11.78 nm^−2^, *n*_[220]_ = 4/(0.585 nm × 0.827 nm × sin (90°)) = 8.26 nm^−2^, and *n*_[222]_ = 4/(0.583 nm × 0.583 nm × sin (120°)) = 13.60 nm^−2^. The density of top layer V atoms of VN is *n* = (*n*_[200]_ + *n*_[220]_ + *n*_[222]_)/3 = 11.21 nm^−2^.

### 4.4. Catalytic Test of Ethane Dehydrogenation

The PSC VN monoliths are used as catalytic reactor. Firstly, the reactor is placed in 5 vol% H_2_/Ar and preheated at 660°C for 2 hours until the residual air is removed. The catalytic test of nonoxidative dehydrogenation of ethane is conducted under normal pressure, while the temperature is increased from 50 to 660°C at a rate of 5°C min^−1^. The catalytic reaction gas contains 10 vol% ethane and balance argon at a flow rate of 5-10 sccm. The products are collected and analyzed using an online gas chromatograph (GC) equipped with FID and TCD detectors (Shimadzu, GC-2014) and a 30 m packed CP-poraplot Q column. The ethane conversion (*X*, %) is determined by

(3) $$\begin{array}{c} X=1-\frac{{\left[2F_{C2H6}\right]}_{\text{out}}}{{\left[2F_{C2H6}\right]}_{\text{out}}+{\left[2F_{C2H4}\right]}_{\text{out}}+{\left[F_{CH4}\right]}_{\text{out}}}\times 100\%. \end{array}$$

The formula of selectivity (*Si*, %) is below, where *Ci* is the concentration (vol.%) of the *i*-th carbon-containing component at the reactor outlet, and *ni* is a coefficient, which takes into account the number of carbon atoms in the products; selectivity to the reaction products (ethylene and carbon oxides) is calculated using

(4) $$\begin{array}{c} Si=\frac{Ci/ni}{\sum \left(Ci/ni\right)}\times 100\%. \end{array}$$

The carbon balance (*Y*, %) is determined by

(5) $$\begin{array}{c} Y=1-\frac{{\left[2F_{C2H6}\right]}_{\text{out}}+{\left[2F_{C2H4}\right]}_{\text{out}}+{\left[F_{CH4}\right]}_{\text{out}}}{{\left[2F_{C2H6}\right]}_{\text{in}}}\times 100\%. \end{array}$$

### 4.5. Theoretical Calculation

Density functional theory (DFT) calculations are executed with Vienna *Ab* Initio Simulation Package (VASP) [23]. The generalized gradient approximation of Perdew-Burke-Ernzerhof (GGA-PBE) is used to represent the exchange-correlation function [24]. And the projector-augmented wave (PAW) method is considered to describe the interaction between the core and valence electrons with a plane wave cut-off energy of 500 eV. The DFT-D3 method [25] and spin-polarization are employed for the calculations with the energy and residual forces converged to 10^−5^ eV and 0.02 eV/Å, respectively. A unit cell of cubic VN with the structural parameters of *a* = *b* = *c* = 4.14 Å is calculated on an 8 × 8 × 8 k-point grid. To avoid the interaction between the slabs, the vacuum region is set at the thickness of 24 Å. A p (3 × 3) superstructure of VN (200) surface is calculated with four layers where the bottom two layers of atoms are frozen. A 3 × 3 × 1 k-point grid is used to sample the Brillouin zone for VN (200) surface. The possible transition state (TS) is performed by climbing image nudged elastic band (CI-NEB) method [26]. We obtain the adsorption energy of C_2_H_6_ using

(6) $$\begin{array}{c} E_{\text{ads}}=E_{\text{total}}-E_{C2H6}-E_{\text{slab}}, \end{array}$$

where *E*_total_ is the total energy of VN (200) surface with adsorbed C_2_H_6_, *E*_C2H6_ is the energy of C_2_H_6_ in free phase, and *E*_slab_ is the energy of the model without any adsorption.

## Data Availability

The data used to support the findings of this study are available from the corresponding author upon request.

## Supplementary Materials

Supplementary Materials Supporting information is available and includes characterization of microstructure and property and theoretical calculation. Figure S1: the structure and characterization of parent single crystals. (a–c) XRD patterns of (001) BiVO_4_, (100) FeVO_4_, and (0-11) CoV_2_O_6_. (d–f) Raman spectra of the parent crystals. (g–i) Mean surface roughness. Figure S2: crystal structure of (001) BiVO_4_ and growth diagram of (220) VN. (a) [001] orientation of BiVO_4_. (b) [001] orientation of BiVO_4_ after the evaporation of Bi. (c) [220] orientation of VN. (d and e) Ball and stick model of the transformation of (001) plane BiVO_4_ to VO_2_ structure. Figure S3: crystal structure of (100) FeVO_4_ and growth diagram of (200) VN. (a) [100] orientation of FeVO_4_ single crystal. (b) [100] orientation of FeVO_4_ single crystal after evaporation of part of Fe atoms. (c) [200] orientation of VN single crystal. (d and e) Ball and stick model of the transformation of (100) plane FeVO_4_ to Fe-doped VO_2_ structure. Figure S4: crystal structure of (0-11) CoV_2_O_6_ and growth diagram of (200) VN. (a) [0-11] orientation of CoV_2_O_6_ single crystal. (b) [0-11] orientation of CoV_2_O_6_ single crystal after evaporation of part of Co atoms. (c) [200] orientation of VN single crystal. (d and e) Ball and stick model of the transformation of (0-11) plane CoV_2_O_6_ to Co-doped VO_2_ structure. Figure S5: energy calculation results. (a-b) The defect energy of Bi during the conversion from (100) BiVO_4_ to (220) VN is 6.35 eV. (a–c) The energy of N-doped formation is 4.96 eV. (d) Simulated diagram of the VN (220) surface. (V in silver, N in blue, Bi in purple, and O in red.) Figure S6: energy calculation results. (a-b) The defect energy of Fe during the conversion from (100) FeVO_4_ to (200) Fe_0.1_V_0.9_N is 3.47 eV. (a–c) The energy of N-doped formation is 3.38 eV. (d) Simulated diagram of the FVN (200) surface. (V in silver, N in blue, Fe in light blue, and O in red.) Figure S7: energy calculation results. (a-b) The defect energy of Co during the conversion from (0-11) CoV_2_O_6_ to (200) Co_0.1_V_0.9_N is 1.92 eV. (a–c) The energy of N-doped formation is 3.05 eV. (d) Simulated diagram of the CVN (200) surface. (V in silver, N in blue, Co in pink, and O in red). Figure S8: porosity characterization of porous VN single crystals and polycrystals. (a) BET specific surface area. (b) BET pore size D50 distribution. (c) Porosity of porous VN, Fe_0.1_V_0.9_N, and Co_0.1_V_0.9_N crystals and polycrystals. Figure S9: ICP and CA tests. Mole ratio between metal and nitrogen in porous VN, FVN, and CVN single crystals and polycrystals. Figure S10: HS-LEISS and visualized structures of VN, FVN, and CVN. HS-LEISS of (a) VN, (d) Fe_0.1_V_0.9_N, and (g) CVN. Structures of (b) VN, (e) FVN, and (h) CVN. The top view of (c) VN, (f) FVN, and (i) Co_0.1_V_0.9_N. (N in blue, V in silver, Fe in light blue, and Co in pink). Figure S11: charge density difference diagrams. (a and d) (220) VN. (b and e) (200) Fe_0.1_V_0.9_N. (c and f) (200) Co_0.1_V_0.9_N. (V in silver, N in blue, Fe in light blue, and Co in pink). Figure S12: comparison of density of states of VN. (a) Density of states of (200) VN and partial density of states of V and N. (b) Density of states of (220) VN and partial density of states of V and N. (c) Density of states of (200) Fe_0.1_V_0.9_N and partial density of states of V, N, and Fe. (d) Density of states of (200) Co_0.1_V_0.9_N and partial density of states of V, N, and Co. Figure S13: the chemical state of the elements. (a and b) XPS of PSC Fe_0.1_V_0.9_N monolith and PSC Co_0.1_V_0.9_N monolith. Figure S14: reaction mechanism of Fe in FVN. (a–d) Wavelet transforms for the *k*^3^-weighted EXAFS signals from sample Fe foil (a) and FVN (b). Figure S15: reaction mechanism of Co in CVN. (a–d) Wavelet transforms for the *k*^3^-weighted EXAFS signals from sample Co foil (a) and CVN (b). Figure S16: characterization of porous VN single crystal structure. (a and b) Cross-sectional STEM image of the pore structure. (c) Cs-HRTEM image of VN crystal. (d–f) Partial enlarged HRTEM image and atomic V and N structure. Bright yellow balls are V atoms, and blue balls are N atoms. Figure S17: the number of V atoms of top surface for unit cell of VN. (a) (200) VN, (b) (220) VN, and (c) (222) VN, respectively. (V in silver and N in blue). Figure S18: microstructure of porous VN polycrystal. (a) XRD pattern of porous VN polycrystal. (b) Cross-sectional STEM image of porous VN polycrystal. (c) HRTEM image of the amorphous layer of VN polycrystal. Figure S19: performance of nonoxidative ethane dehydrogenation with porous VN polycrystals. (a) The ethane conversion of porous VN polycrystals. (b) The ethylene selectivity of porous VN polycrystals. Figure S20: comparison of crystal structure before and after stability test. (a–c) The XRD spectra of the catalysts before the stability test. (d–f) The XRD spectra of the catalysts after the stability test. Figure S21: characterization of Raman spectroscopy. (a–c) The Raman spectra of the catalysts before the stability test. (d–f) The Raman spectra of the catalysts after the stability test. Figure S22: HS-LEIS spectra after the stability test of ethane dehydrogenation. (a–c) HS-LEIS spectra with 3 KeV He^+^ as ion source of PSC VN, Fe_0.1_V_0.9_N, and Co_0.1_V_0.9_N, respectively. (d–f) HS-LEIS spectra with 5 KeV Ne^+^ as ion source of PSC VN, Fe_0.1_V_0.9_N, and Co_0.1_V_0.9_N, respectively. Figure S23: SEM of PSC monoliths after operation. (a–c) SEM of PSC VN, Fe_0.1_V_0.9_N, and Co_0.1_V_0.9_N monoliths after continuous operation of 200 hours using the atmosphere of 10% C_2_H_6_/90% Ar, respectively. Table S1: result of the fit performed on the EXAFS spectrum of Fe_0.1_V_0.9_N and Co_0.1_V_0.9_N.
