# Supplementary material for: Porous Single-Crystalline Monolith to Enhance Catalytic Activity and Stability
Source: Research (Wash D C). 2022 Jul 5;2022:9861518. doi: 10.34133/2022/9861518 (PMC9297723; doi:10.34133/2022/9861518)
Supplement: Supplementary Materials — Supporting information is available and includes characterization of microstructure and property and theoretical calculation. Figure S1: the structure and characterization of parent single crystals. (a–c) XRD patterns of (001) BiVO4, (100) FeVO4, and (0-11) CoV2O6. (d–f) Raman spectra of the parent crystals. (g–i) Mean surface roughness. Figure S2: crystal structure of (001) BiVO4 and growth diagram of (220) VN. (a) [001] orientation of BiVO4. (b) [001] orientation of BiVO4 after the evaporation of Bi. (c) [220] orientation of VN. (d and e) Ball and stick model of the transformation of (001) plane BiVO4 to VO2 structure. Figure S3: crystal structure of (100) FeVO4 and growth diagram of (200) VN. (a) [100] orientation of FeVO4 single crystal. (b) [100] orientation of FeVO4 single crystal after evaporation of part of Fe atoms. (c) [200] orientation of VN single crystal. (d and e) Ball and stick model of the transformation of (100) plane FeVO4 to Fe-doped VO2 structure. Figure S4: crystal structure of (0-11) CoV2O6 and growth diagram of (200) VN. (a) [0-11] orientation of CoV2O6 single crystal. (b) [0-11] orientation of CoV2O6 single crystal after evaporation of part of Co atoms. (c) [200] orientation of VN single crystal. (d and e) Ball and stick model of the transformation of (0-11) plane CoV2O6 to Co-doped VO2 structure. Figure S5: energy calculation results. (a-b) The defect energy of Bi during the conversion from (100) BiVO4 to (220) VN is 6.35 eV. (a–c) The energy of N-doped formation is 4.96 eV. (d) Simulated diagram of the VN (220) surface. (V in silver, N in blue, Bi in purple, and O in red.) Figure S6: energy calculation results. (a-b) The defect energy of Fe during the conversion from (100) FeVO4 to (200) Fe0.1V0.9N is 3.47 eV. (a–c) The energy of N-doped formation is 3.38 eV. (d) Simulated diagram of the FVN (200) surface. (V in silver, N in blue, Fe in light blue, and O in red.) Figure S7: energy calculation results. (a-b) The defect energy of Co during th [file 9861518.f1.docx]

**Supporting Information**

**Porous Single-Crystalline Monolith to Enhance Catalytic Activity and Stability**

Xiaoyan Yu,^1†^ Fangyuan Cheng,^1,2†^ Xiuyun Duan,^1,2†^ Kui Xie^1,2,3,4*^

^1^ Key Laboratory of Optoelectronic Materials Chemistry and Physics, Fujian Institute of Research on the Structure of Matter, Chinese Academy of Sciences, Fuzhou, Fujian 350002, China.

^2^ University of Chinese Academy of Sciences, Beijing 100049, China.

^3^ Fujian Science & Technology Innovation Laboratory for Optoelectronic Information of China, Fuzhou 350108, Fujian, China.

^4^ Key Laboratory of Design & Assembly of Functional Nanostructures, Fujian Institute of Research on the Structure of Matter, Chinese Academy of Sciences, Fuzhou, Fujian 350002, China.

*Correspondence should be addressed to Kui Xie; [kxie@fjirsm.ac.cn](mailto:kxie@fjirsm.ac.cn)

†These authors contributed equally.

Supplementary Figures.

**
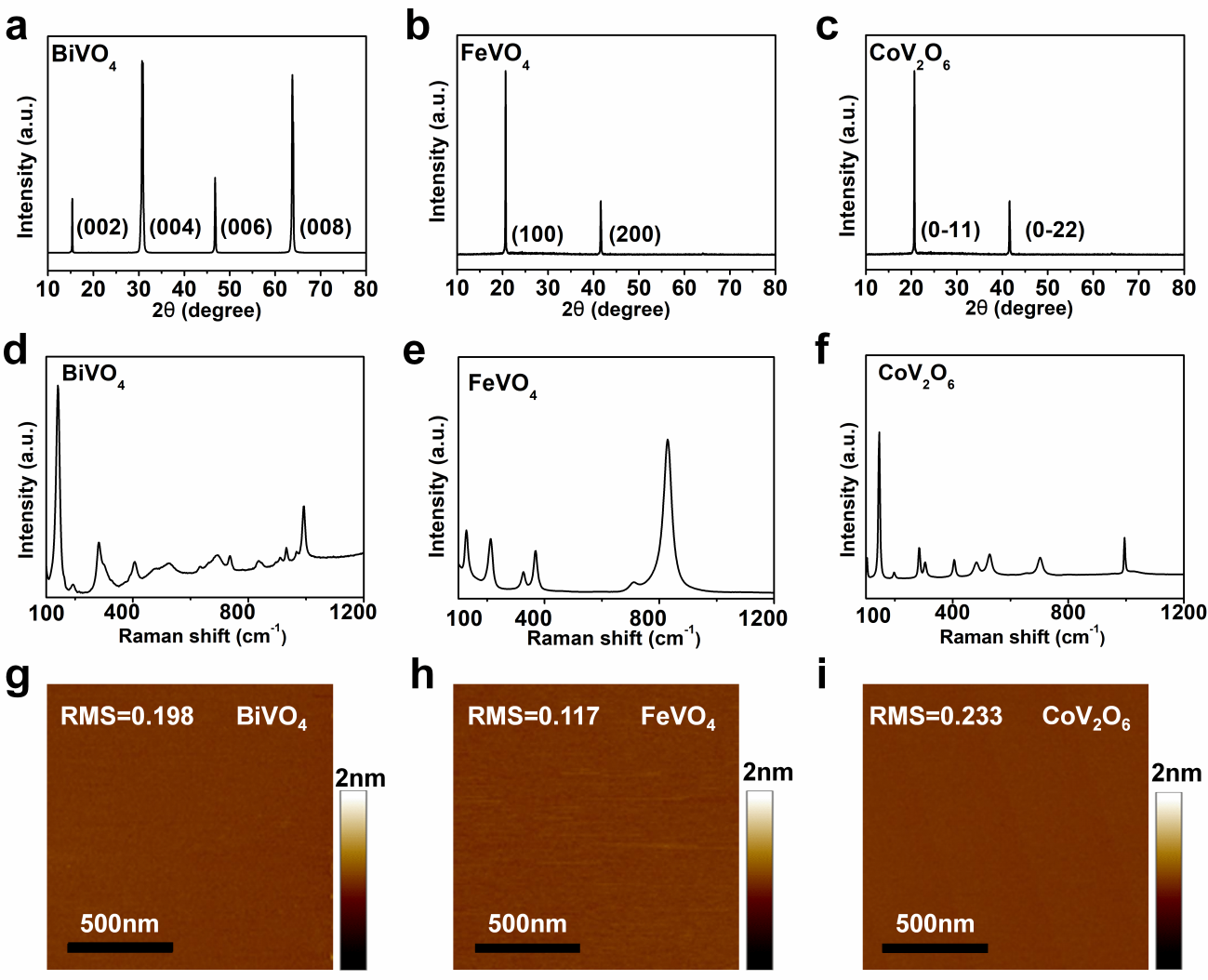
**

**Figure S1: The structure and characterization of parent single crystals.** (a-c) XRD patterns of (001) BiVO_4_, (100) FeVO_4_ and (0-11) CoV_2_O_6_. (d-f) Raman spectra of the parent crystals. (g-i) Mean surface roughness.


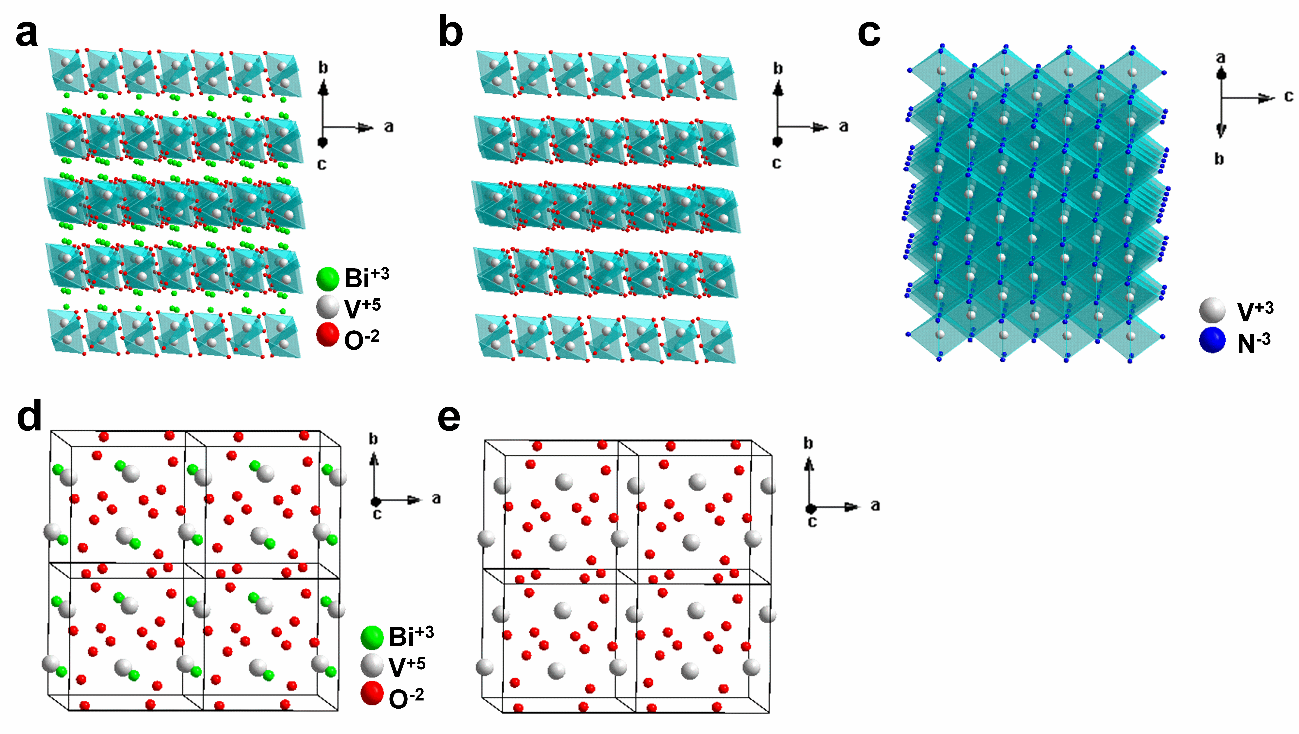


**Figure S2: Crystal structure of (001) BiVO_4_ and growth diagram of (220) VN.** (a) [001] orientation of BiVO_4_. (b) [001] orientation of BiVO_4_ after the evaporation of Bi. (c) [220] orientation of VN. (d and e) Ball and stick model of the transformation of (001) plane BiVO_4_ to VO_2_ structure.


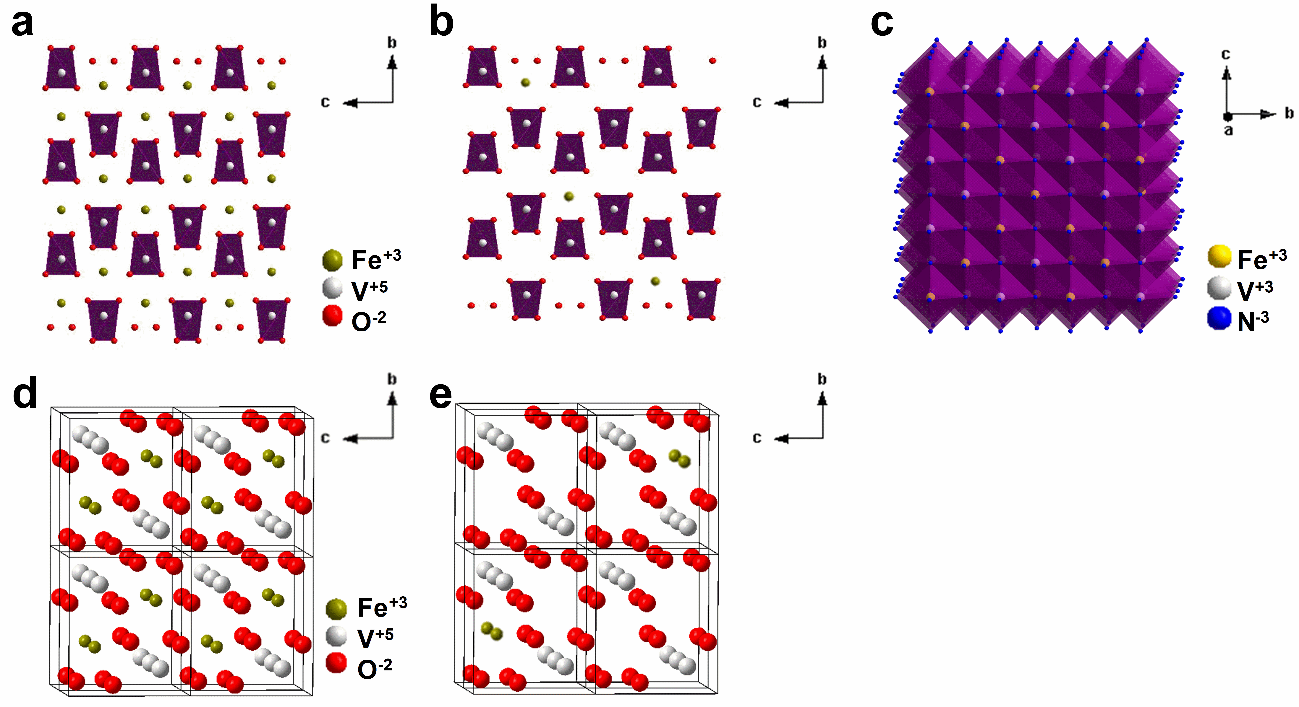


**Figure S3: Crystal structure of (100) FeVO_4_ and growth diagram of (200) VN.** (a) [100] orientation of FeVO_4_ single crystal. (b) [100] Orientation of FeVO_4_ single crystal after evaporation of part of Fe atoms. (c) [200] orientation of VN single crystal. (d and e) Ball and stick model of the transformation of (100) plane FeVO_4_ to Fe-doped VO_2_ structure.


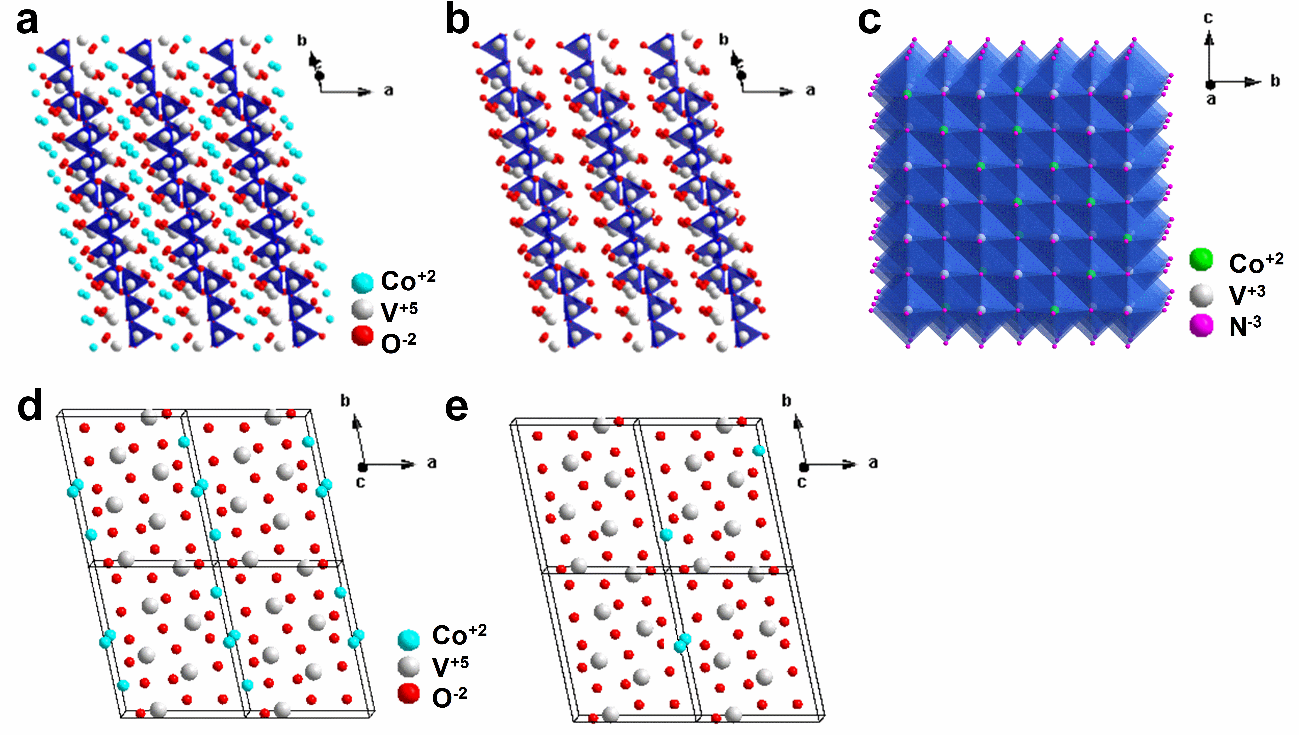


**Figure S4:** **Crystal structure of (0-11) CoV_2_O_6_ and growth diagram of (200) VN.** (a) [0-11] orientation of CoV_2_O_6_ single crystal. (b) [0-11] Orientation of CoV_2_O_6_ single crystal after evaporation of part of Co atoms. (c) [200] orientation of VN single crystal. (d and e) Ball and stick model of the transformation of (0-11) plane CoV_2_O_6_ to Co-doped VO_2_ structure.


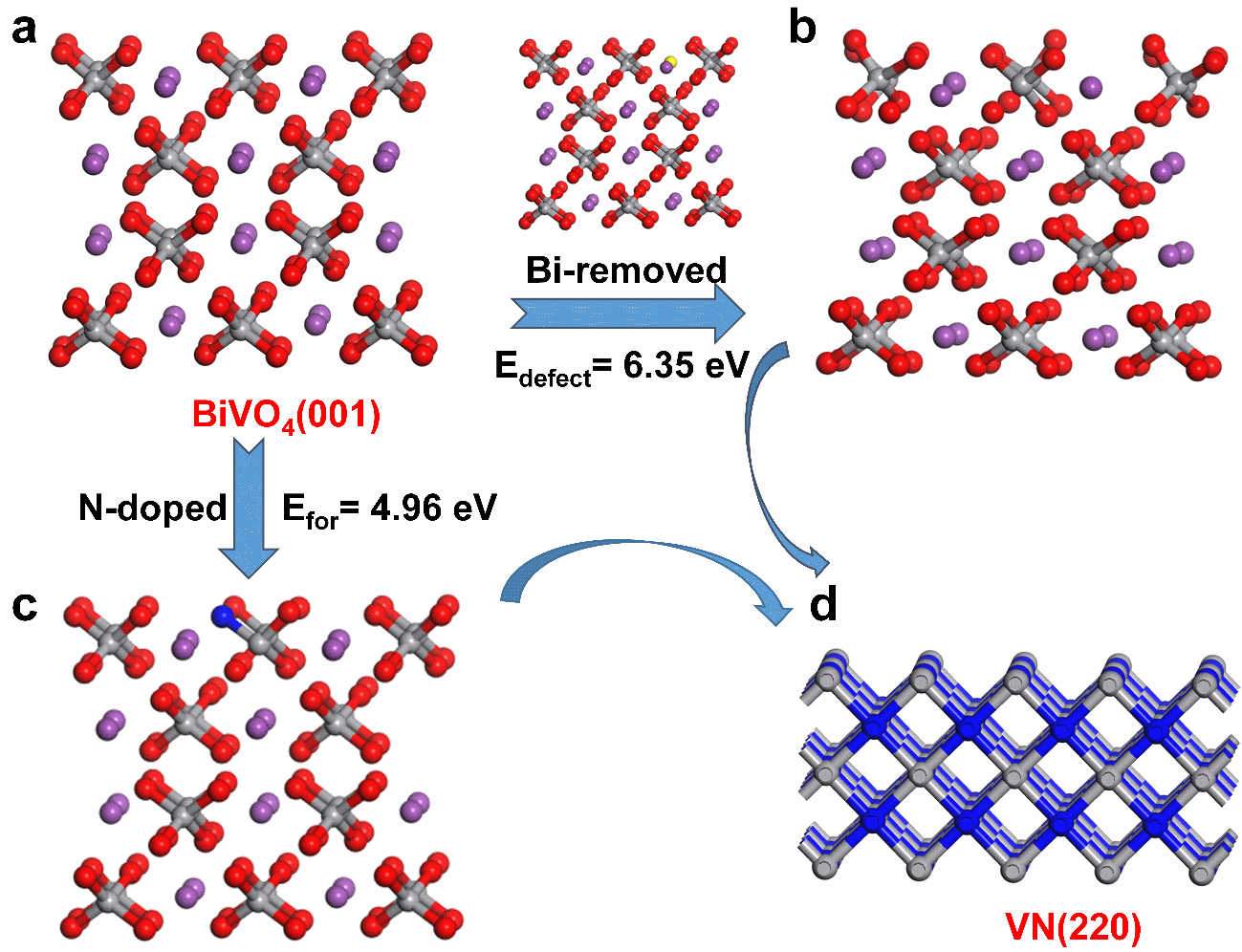


**Figure S5: Energy calculation results.** (a-b) The defect energy of Bi during the conversion from (100) BiVO_4_ to (220) VN is 6.35 eV. (a-c) The energy of N-doped formation is 4.96 eV. (d) Simulated diagram of the VN (220) surface. (V in sliver, N in blue, Bi in purple and O in red.)


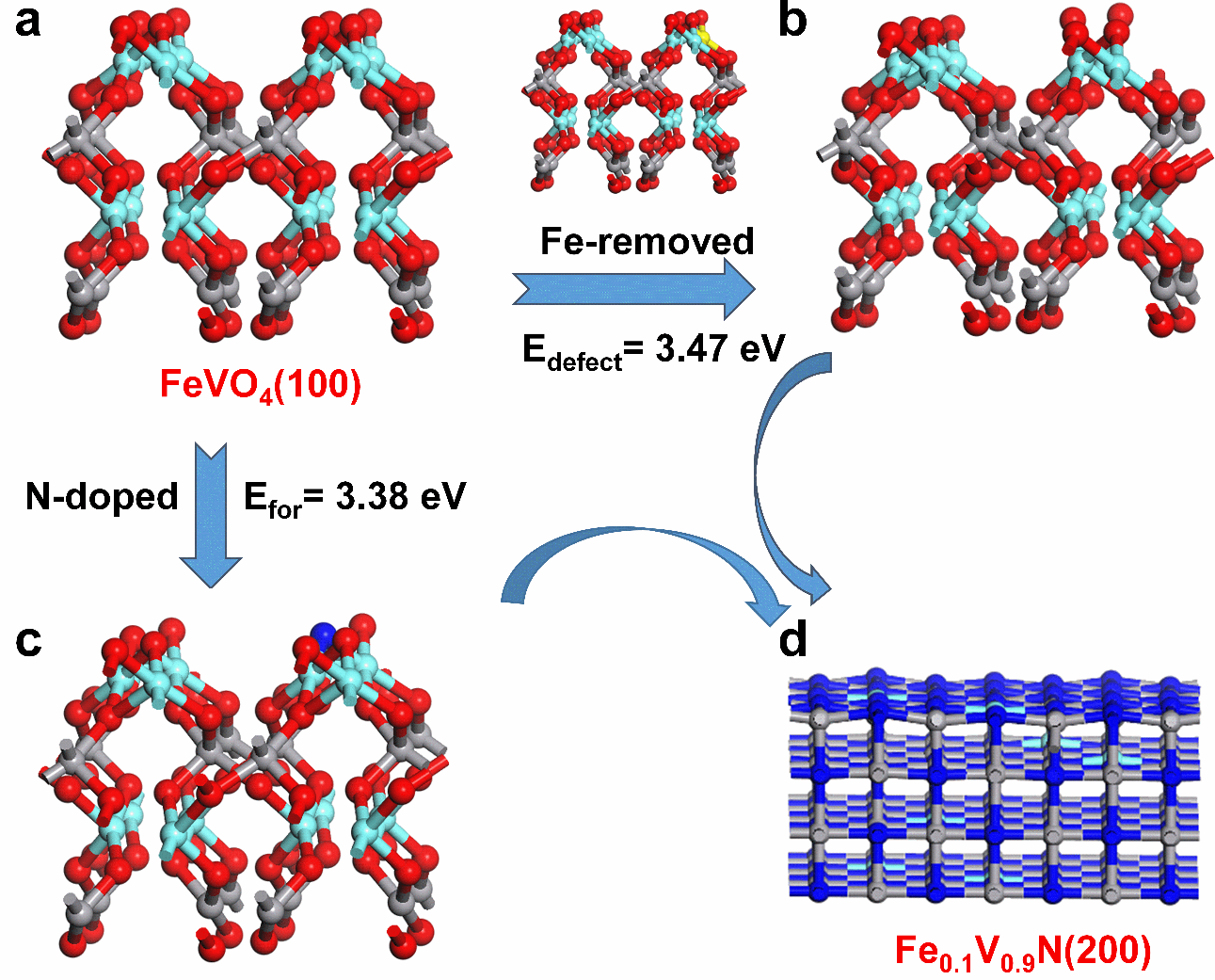


**Figure S6: Energy calculation results.** (a-b) The defect energy of Fe during the conversion from (100) FeVO_4_ to (200) Fe_0.1_V_0.9_N is 3.47 eV. (a-c) The energy of N-doped formation is 3.38 eV. (d) Simulated diagram of the FVN (200) surface. (V in sliver, N in blue, Fe in light blue and O in red.)


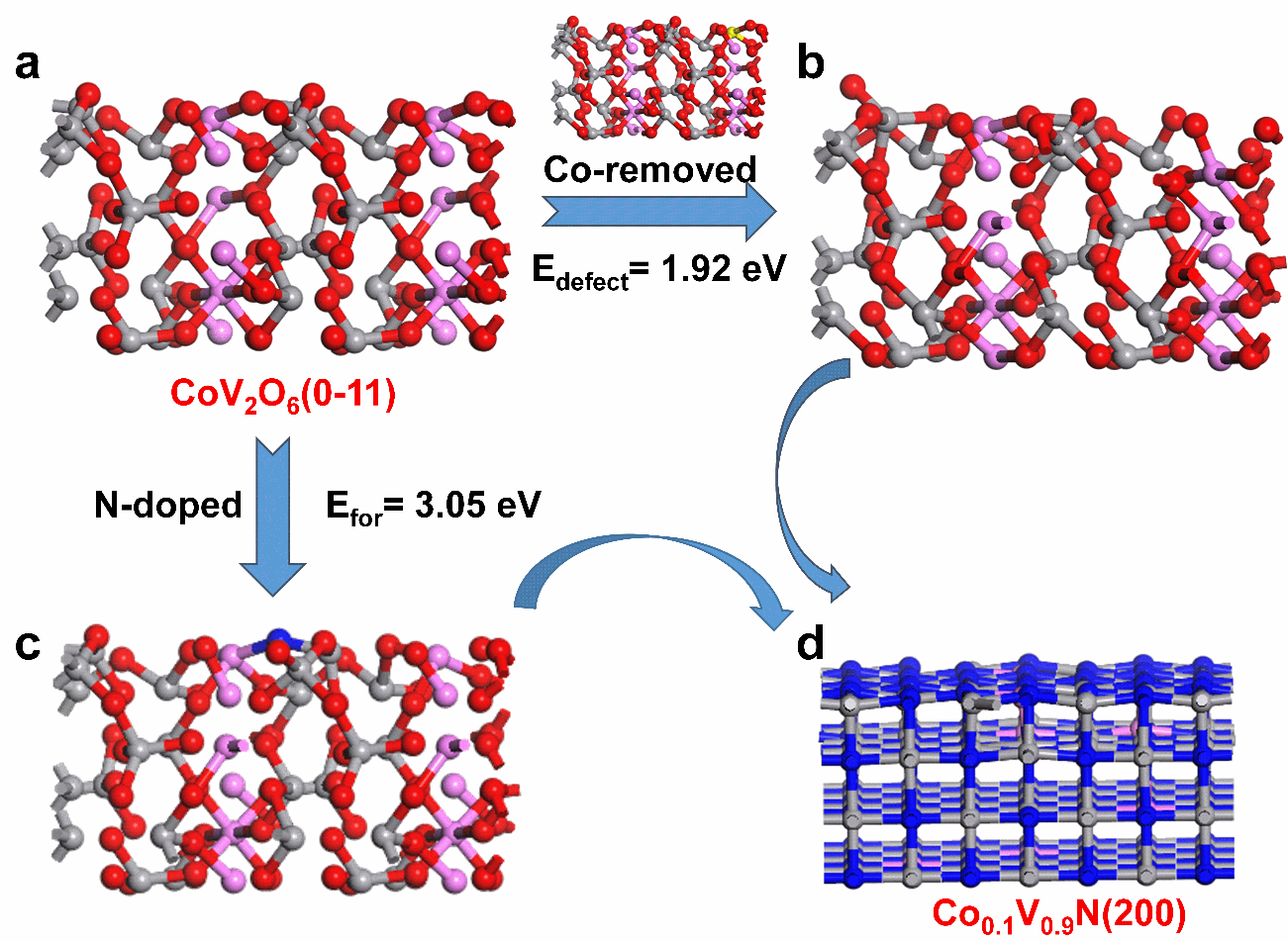


**Figure S7: Energy calculation results.** (a-b) The defect energy of Co during the conversion from (0-11) CoV_2_O_6_ to (200) Co_0.1_V_0.9_N is 1.92 eV. (a-c) The energy of N-doped formation is 3.05 eV. (d) Simulated diagram of the CVN (200) surface. (V in sliver, N in blue, Co in pink and O in red).


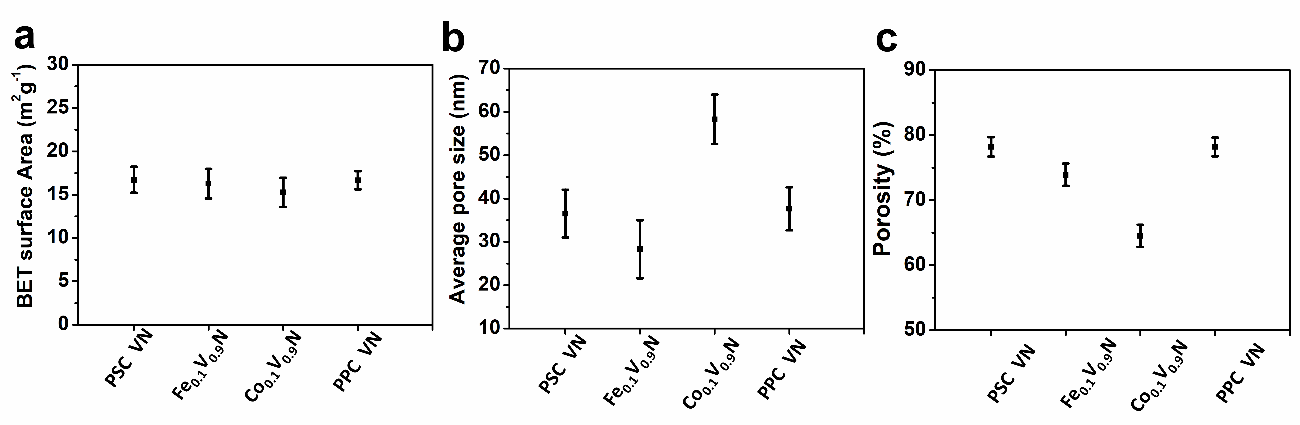


**Figure S8: Porosity characterization of porous VN single crystals and polycrystals.** (a) BET specific surface area. (b) BET pore size D50 distribution. (c) Porosity of porous VN, Fe_0.1_V_0.9_N and Co_0.1_V_0.9_N crystals and polycrystals.


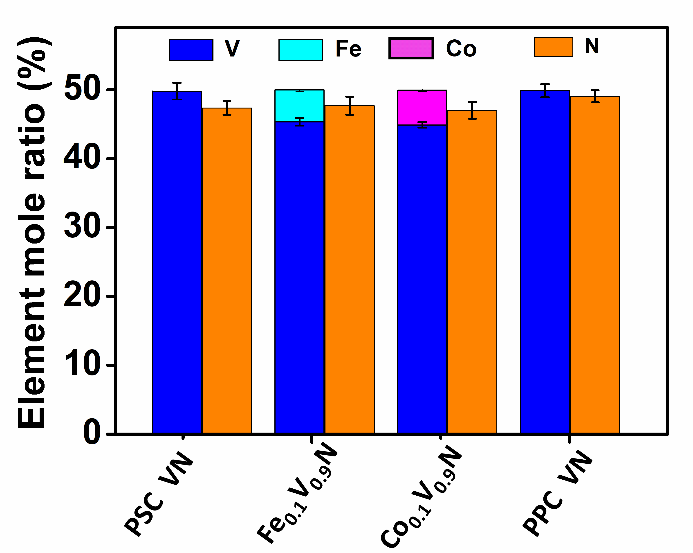


**Figure S9:** **ICP and CA tests.** Mole ratio between metal and nitrogen in porous VN, FVN and CVN single crystals and polycrystals.


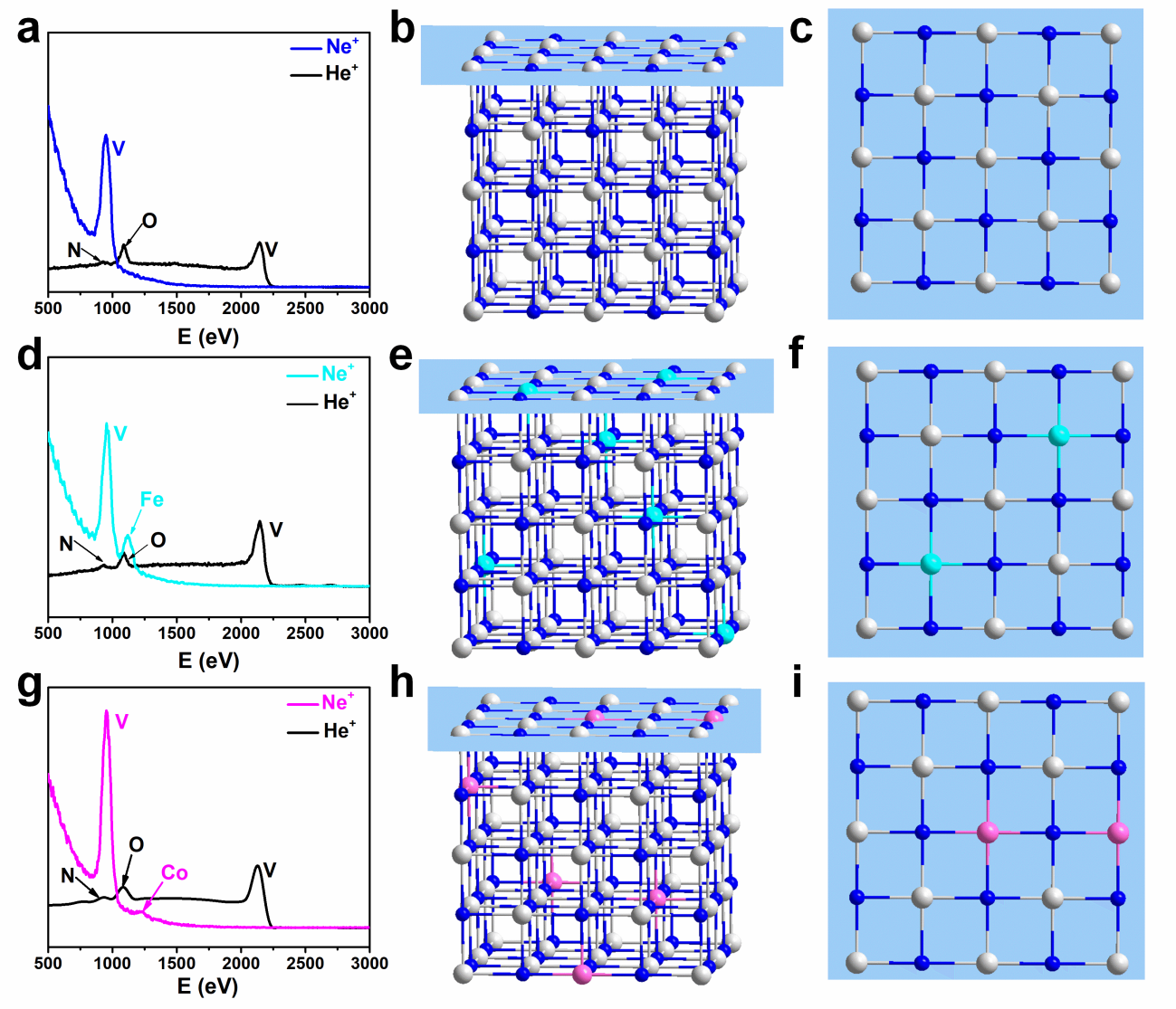


**Figure S10: HS-LEISS and visualized structures of VN, FVN and CVN.** HS-LEISS of (a) VN, (d) FVN, (g) CVN. Structures of (b) VN, (e) FVN and (h) CVN. The top view of (c) VN, (f) FVN and (i) CVN. (N in blue, V in sliver, Fe in light blue and Co in pink)


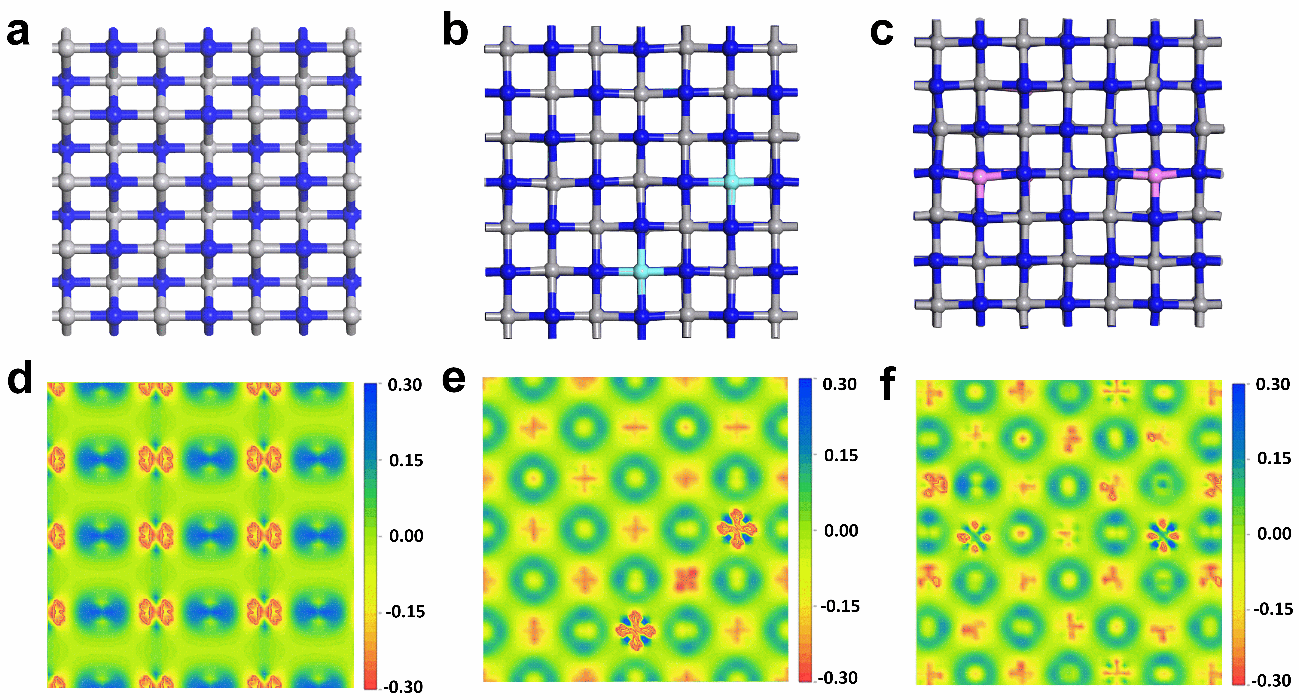


**Figure S11: Charge density difference diagrams.** (a and d) (220) VN. (b and e) (200) Fe_0.1_V_0.9_N. (c and f) (200) Co_0.1_V_0.9_N. (V in sliver, N in blue, Fe in light blue, Co in pink).


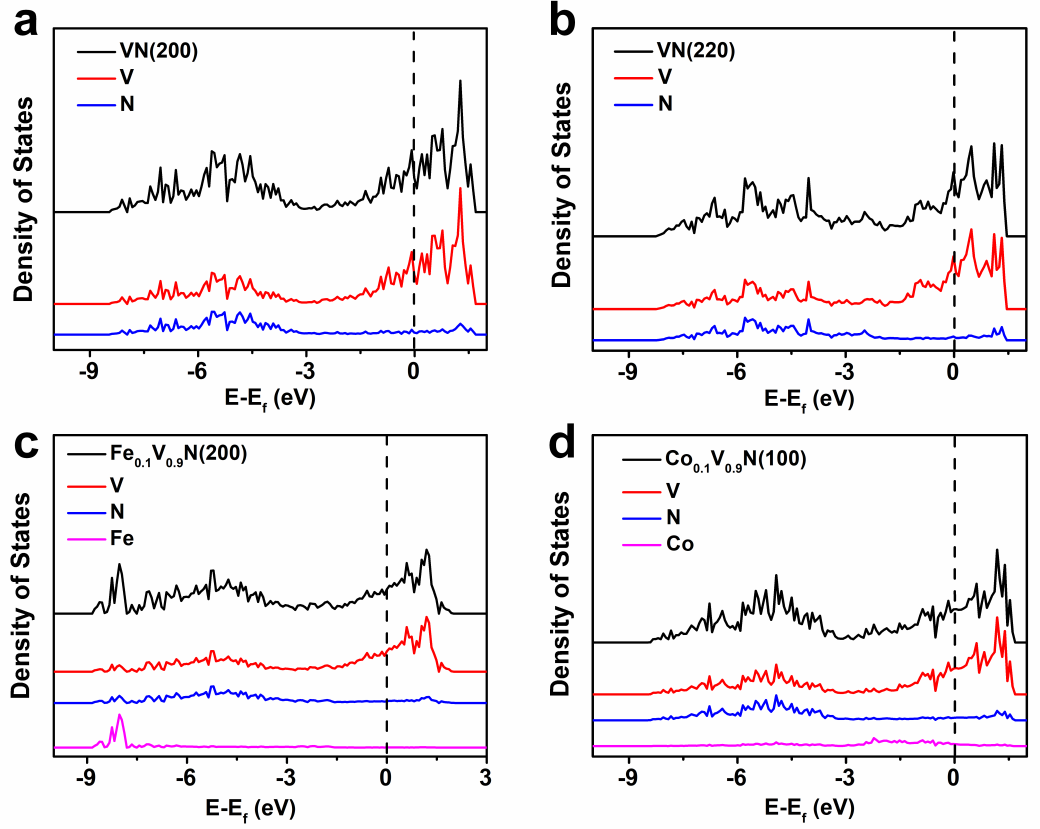


**Figure S12: Comparison of density of states of VN.** (a) Density of states of (200) VN and partial density of states of V and N. (b) Density of states of (220) VN and partial density of states of V and N. (c) Density of states of (200) Fe_0.1_V_0.9_N and partial density of states of V, N and Fe. (d) Density of states of (200) Co_0.1_V_0.9_N and partial density of states of V, N and Co.


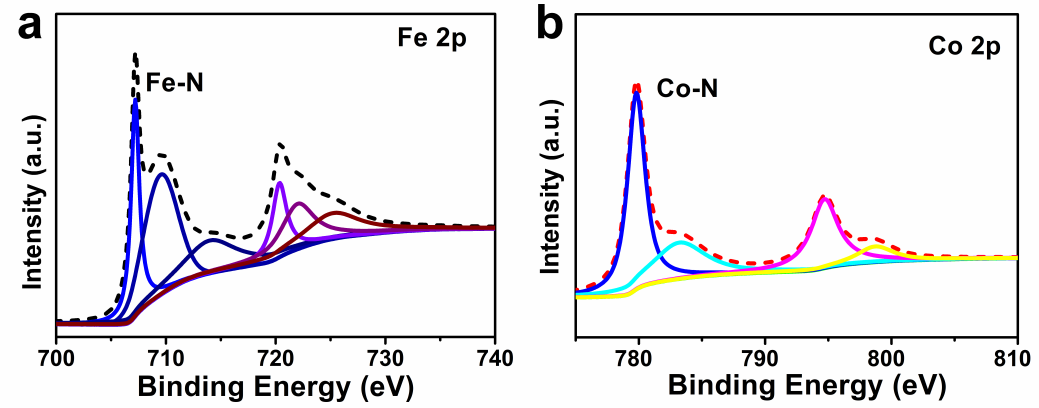


**Figure S13: The chemical state of the elements**. (a and b) XPS of PSC Fe_0.1_V_0.9_N monolith and PSC Co_0.1_V_0.9_N monolith.


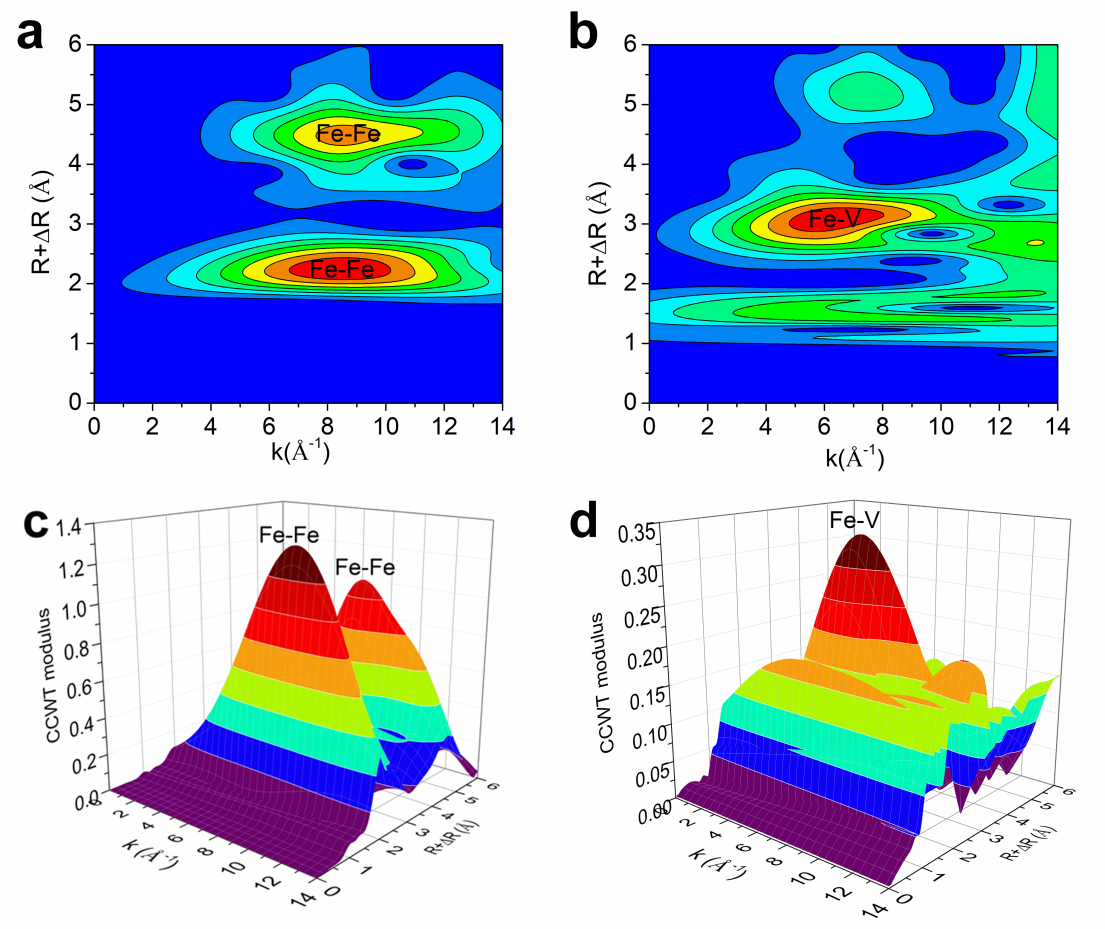


**Figure S14:** **Reaction mechanism of Fe in FVN.** (a-d) Wavelet transforms for the k^3^-weighted EXAFS signals from sample Fe foil (a) and FVN (b).


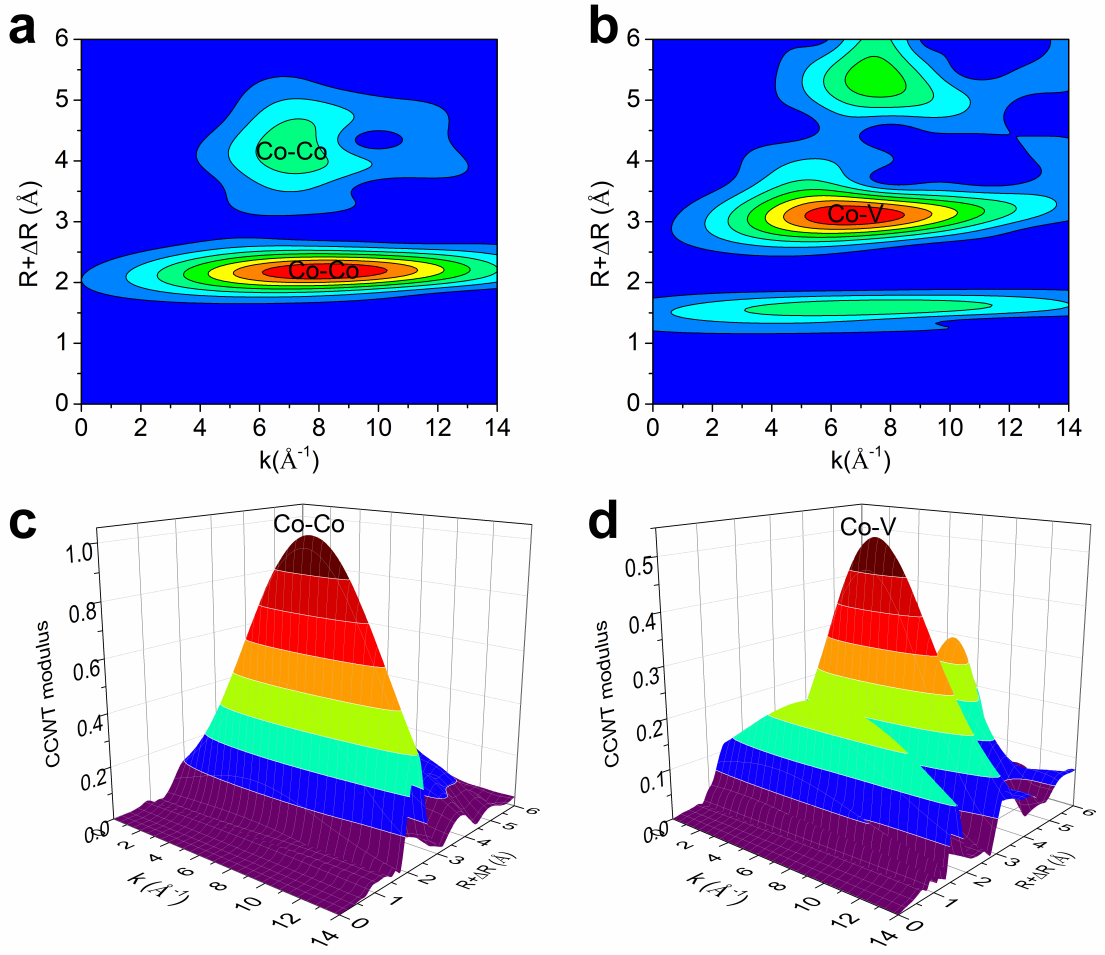


**Figure S15:** **Reaction mechanism of Co in CVN.** (a-d) Wavelet transforms for the k^3^-weighted EXAFS signals from sample Co foil (a) and CVN (b).


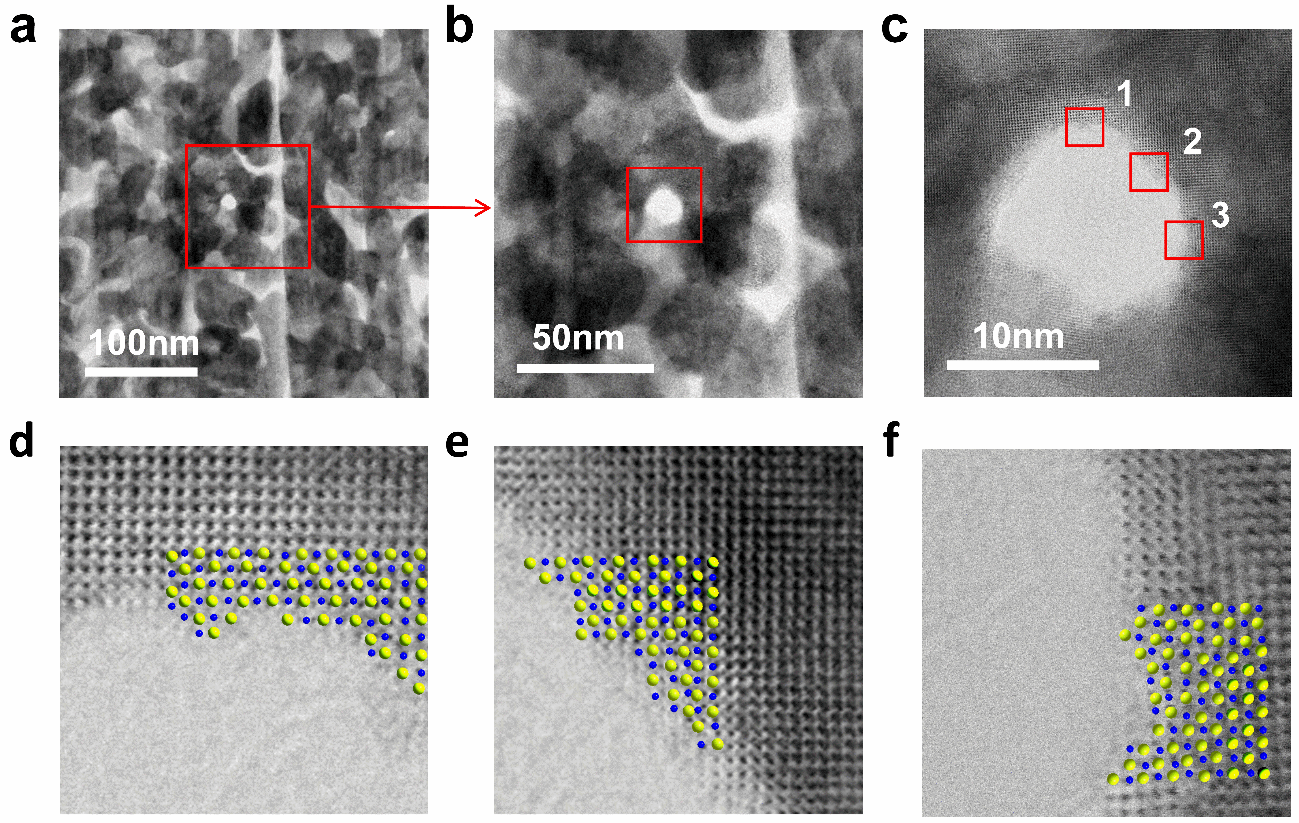


**Figure S16: Characterization of porous VN single crystal structure.** (a and b) Cross-sectional STEM image of the pore structure. (c) Cs-HRTEM image of VN crystal. (d-f) Partial enlarged HRTEM image and atomic V and N structure. Bright yellow balls are V atoms, and blue balls are N atoms.


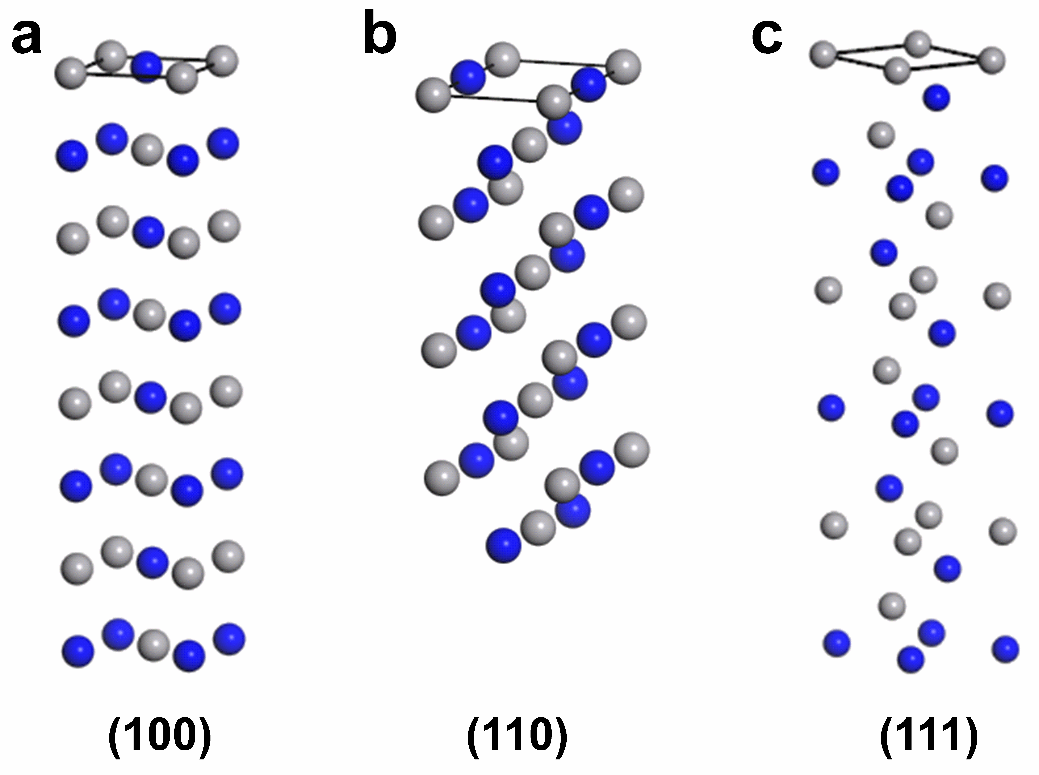


**Figure S17: The number of V atoms of top surface for unit cell of VN.** (a) (200) VN. (b) (220) VN and (c) (222) VN, respectively. (V in silver, N in blue).


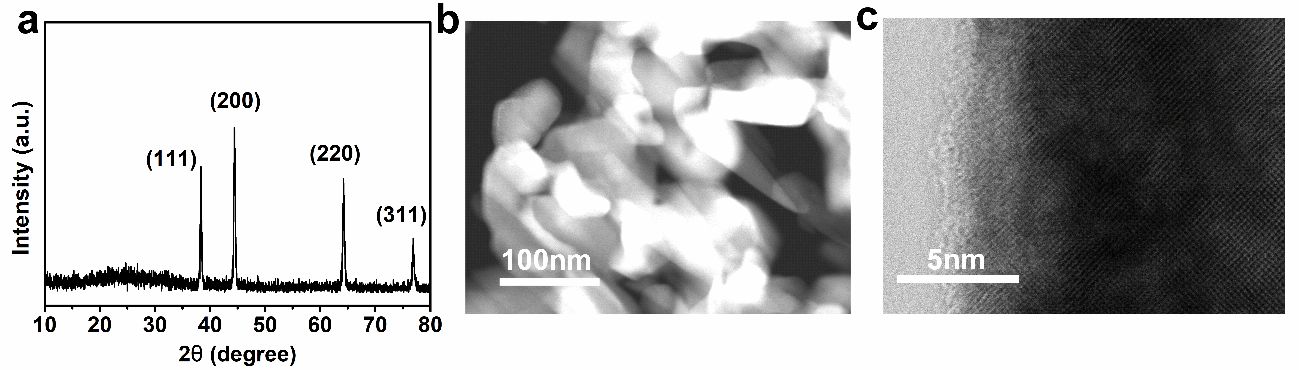


**Figure 18: Microstructure of porous VN polycrystal.** (a) XRD pattern of porous VN polycrystal. (b) Cross-sectional STEM image of porous VN polycrystal. (c) HRTEM image of the amorphous layer of VN polycrystal.


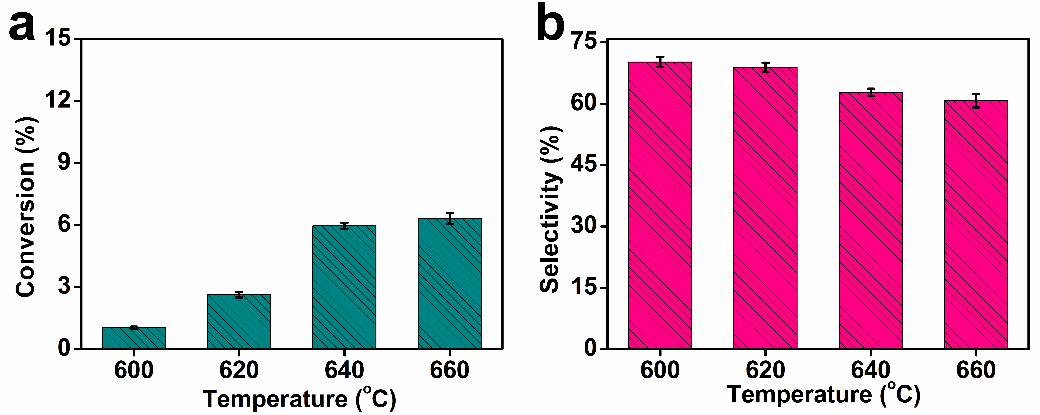


**Figure S19: Performance of nonoxidative ethane dehydrogenation with porous VN polycrystals.** (a) The ethane conversion of porous VN polycrystals. (b) The ethylene selectivity of porous VN polycrystals.


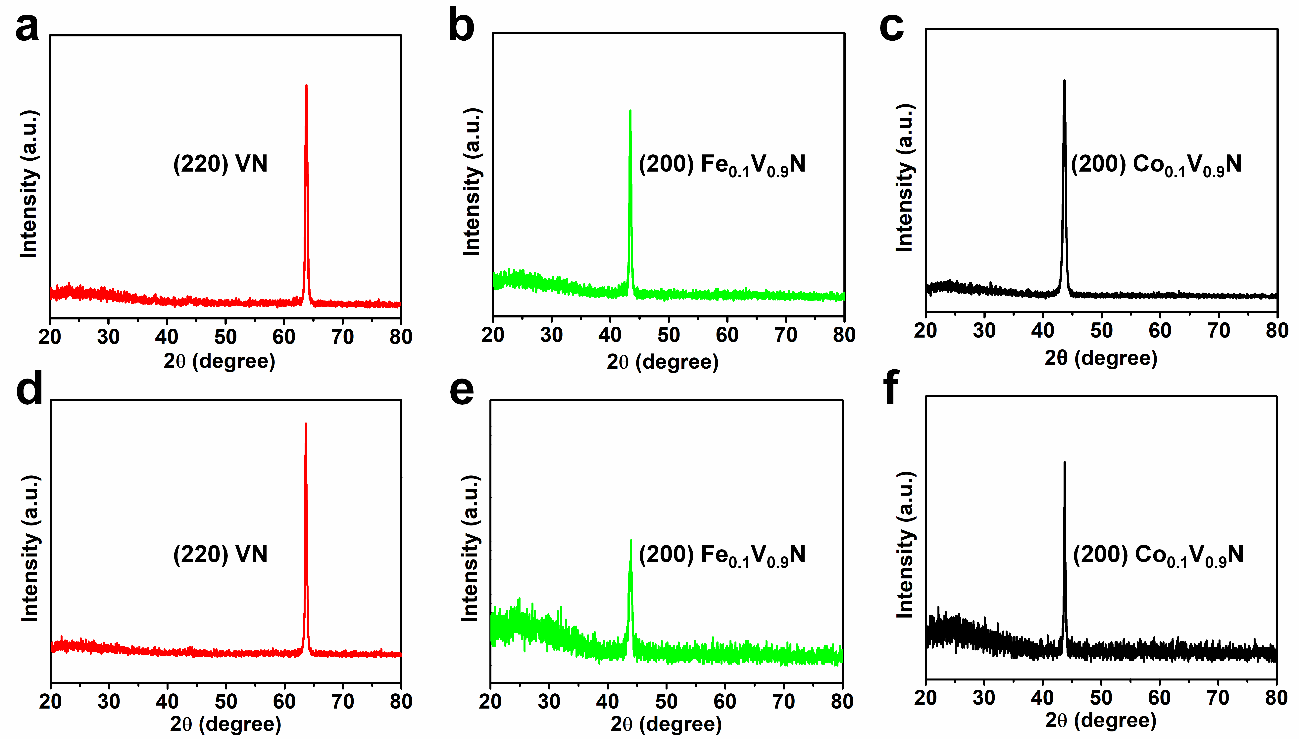


**Figure S20: Comparison of crystal structure before and after stability test.** (a-c) The XRD spectra of the catalysts before the stability test. (d-f) The XRD spectra of the catalysts after the stability test.


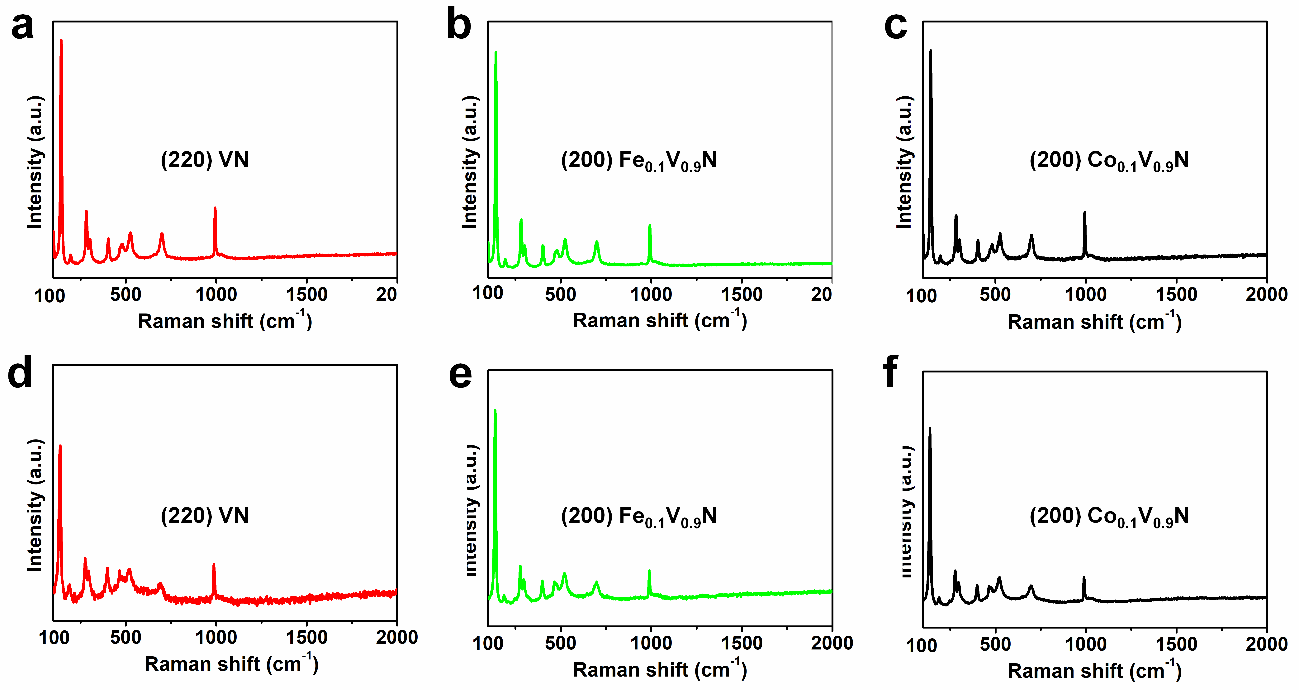


**Figure S21: Characterization of Raman spectroscopy.** (a-c) The Raman spectra of the catalysts before the stability test. (d-f) The Raman spectra of the catalysts after the stability test.


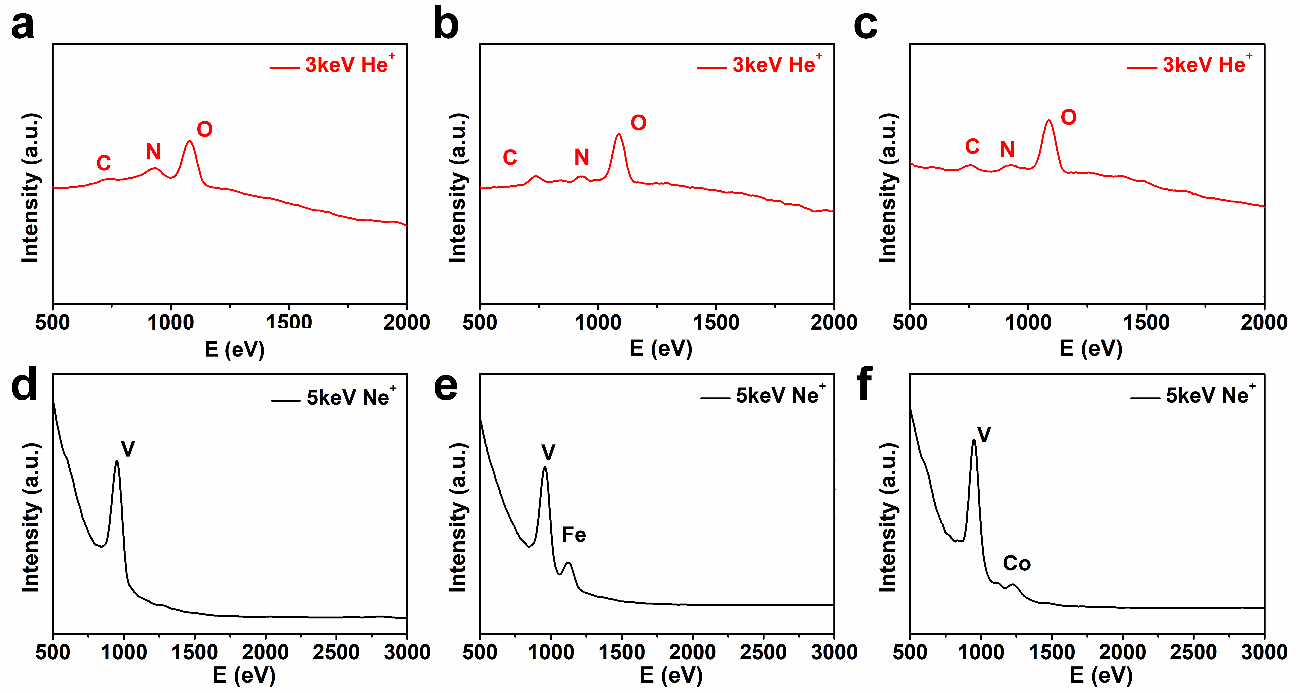


**Figure S22: HS-LEIS spectra after the stability test of ethane dehydrogenation.** (a-c) HS-LEIS spectra with 3 KeV He^+^ as ion source of PSC VN, Fe_0.1_V_0.9_N and Co_0.1_V_0.9_N, respectively. (d-f) HS-LEIS spectra with 5 KeV Ne^+^ as ion source of PSC VN, Fe_0.1_V_0.9_N and Co_0.1_V_0.9_N, respectively.


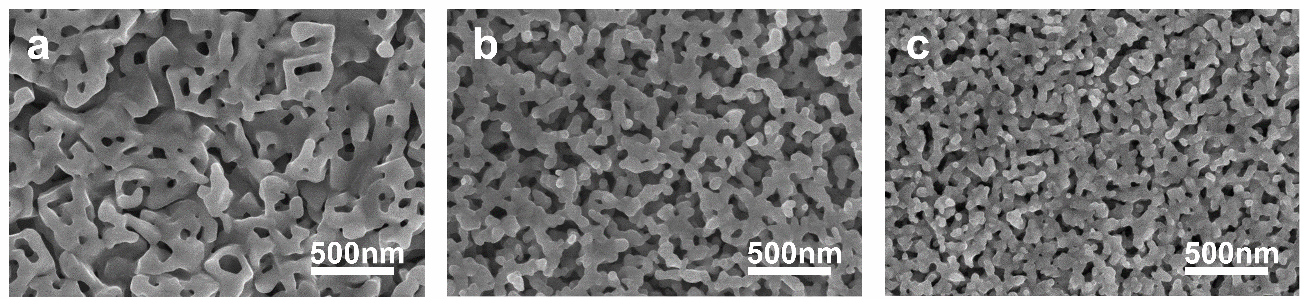


**Figure S23: SEM of PSC monoliths after operation.** (a-c) SEM of PSC VN, Fe_0.1_V_0.9_N and Co_0.1_V_0.9_N monoliths after continuous operation of 200 hours using the atmosphere of 10% C_2_H_6_/90% Ar, respectively.

**Table S1: Result of the fit performed on the EXAFS spectrum of Fe_0.1_V_0.9_N and Co_0.1_V_0.9_N.**

| Sample | Path | CN | σ^2^(*10^-2^Å^-2^) | R(Å) | △E0(eV) | R factor |
| --- | --- | --- | --- | --- | --- | --- |
| Co-VN | Co-N | 3.622±0.259 | 0.277±0.105 | 1.756±0.0056 | 11.017±0.516 | 0.0068 |
|  | Co-V | 13.831±1.087 | 0.972±0.094 | 3.269±0.0065 |  |  |
| Fe-VN | Fe-N | 4.109±0.943 | 0.613±0.451 | 1.566±0.020 | 9.412±1.789 | 0.0016 |
|  | Fe-V | 9.758±2.922 | 0.953±0.399 | 3.188±0.0227 |  |  |
